# Ancient female philopatry, asymmetric male gene flow, and synchronous population expansion support the influence of climatic oscillations on the evolution of South American sea lion (*Otaria flavescens*)

**DOI:** 10.1371/journal.pone.0179442

**Published:** 2017-06-27

**Authors:** Larissa Rosa de Oliveira, Marcelo C. M. Gehara, Lúcia D. Fraga, Fernando Lopes, Juan Ignacio Túnez, Marcelo H. Cassini, Patricia Majluf, Susana Cárdenas-Alayza, Héctor J. Pavés, Enrique Alberto Crespo, Nestor García, Rocío Loizaga de Castro, A. Rus Hoelzel, Maritza Sepúlveda, Carlos Olavarría, Victor Hugo Valiati, Renato Quiñones, Maria Jose Pérez-Alvarez, Paulo Henrique Ott, Sandro L. Bonatto

**Affiliations:** 1 Laboratório de Ecologia de Mamíferos, Universidade do Vale do Rio dos Sinos, São Leopoldo, RS, Brazil; 2 Grupo de Estudos de Mamíferos Aquáticos do Rio Grande do Sul, Osório, RS, Brazil; 3 Herpetology, American Museum of Natural History, New York, NY, United States of America; 4 Faculdade de Biociências, Pontifícia Universidade Católica do Rio Grande do Sul, Porto Alegre, RS, Brazil; 5 Grupo GEMA, Departamento de Ciencias Básicas, Universidad Nacional de Luján and CONICET, Luján, Buenos Aires, Argentina; 6 Laboratorio de Biología del Comportamiento, Instituto de Biología y Medicina Experimental (CONICET), Buenos Aires, Argentina; 7 Centro para la Sostenibilidad Ambiental / Universidad Peruana Cayetano Heredia, Lima, Perú; 8 Departamento de Ciencias Básica, Facultad de Ciencias, Universidad Santo Tomas, Osorno, Chile; 9 Laboratorio de Mamíferos Marinos, Centro para el Estudio de los Sistemas Marinos (CENPAT-CONICET), Puerto Madryn, Chubut, Argentina; 10 Department of Biosciences, Durham University, Durham, United Kingdom; 11 Centro de Investigación y Gestión de los Recursos Naturales, Instituto de Biología, Facultad de Ciencias, Universidad de Valparaíso, Valparaíso, Chile; 12 Millenium Nucleus of Invasive Salmonids (INVASAL), Concepción, Chile; 13 Centro de Estudios Avanzados en Zonas Aridas, La Serena, Chile; 14 Laboratório de Biologia Molecular, Universidade do Vale do Rio dos Sinos, São Leopoldo, RS, Brazil; 15 Interdisciplinary Center for Aquaculture Research (FONDAP), Universidad de Concepción, Concepción, Chile; 16 Instituto de Ecología y Biodiversidad, Facultad de Ciencias, Universidad de Chile, Santiago, Chile; 17 Laboratório de Ecologia e Conservação de Organismos Aquáticos, Universidade Estadual do Rio Grande do Sul, Osório, RS, Brazil; National Cheng Kung University, TAIWAN

## Abstract

The South American sea lion (*Otaria flavescens*) is widely distributed along the southern Atlantic and Pacific coasts of South America with a history of significant commercial exploitation. We aimed to evaluate the population genetic structure and the evolutionary history of South American sea lion along its distribution by analyses of mitochondrial DNA (mtDNA) and 10 nuclear microsatellites loci. We analyzed 147 sequences of mtDNA control region and genotyped 111 individuals of South American sea lion for 10 microsatellite loci, representing six populations (Peru, Northern Chile, Southern Chile, Uruguay (Brazil), Argentina and Falkland (Malvinas) Islands) and covering the entire distribution of the species. The mtDNA phylogeny shows that haplotypes from the two oceans comprise two very divergent clades as observed in previous studies, suggesting a long period (>1 million years) of low inter-oceanic female gene flow. Bayesian analysis of bi-parental genetic diversity supports significant (but less pronounced than mitochondrial) genetic structure between Pacific and Atlantic populations, although also suggested some inter-oceanic gene flow mediated by males. Higher male migration rates were found in the intra-oceanic population comparisons, supporting very high female philopatry in the species. Demographic analyses showed that populations from both oceans went through a large population expansion ~10,000 years ago, suggesting a very similar influence of historical environmental factors, such as the last glacial cycle, on both regions. Our results support the proposition that the Pacific and Atlantic populations of the South American sea lion should be considered distinct evolutionarily significant units, with at least two managements units in each ocean.

## Introduction

In the marine environment, there is considerable potential for connectivity and dispersion, and barriers to dispersal can be difficult to recognize. This is especially true for highly mobile species such as marine mammals and pelagic fish. While some of these species do appear to show panmixia across vast oceanographic scales (e.g. the European eel, *Anguilla Anguilla* [1]; the blue hake, *Antimora rostrata* [2]; and the common dolphin, *Delphinus delphis* in the North Atlantic [3]), it is more common for population subdivision to be found. This is especially true for cetacean species, sometimes over relatively smaller geographic scales [4] and the challenge is to understand the relevant processes. In some cases, the boundaries to gene flow appear to be associated with habitat or resource specializations [5]. In at least one case strong differentiation between parapatric forms of dolphins in the genus *Delphinus* appears to have evolved during a transitional period of climate change when novel habitat was released after the last ice age due to changing current patterns [6]. Here we consider the biogeographic mechanisms that may define population structure in another coastal marine mammal species, distributed around the southern end of South America.

**Fig 1:**
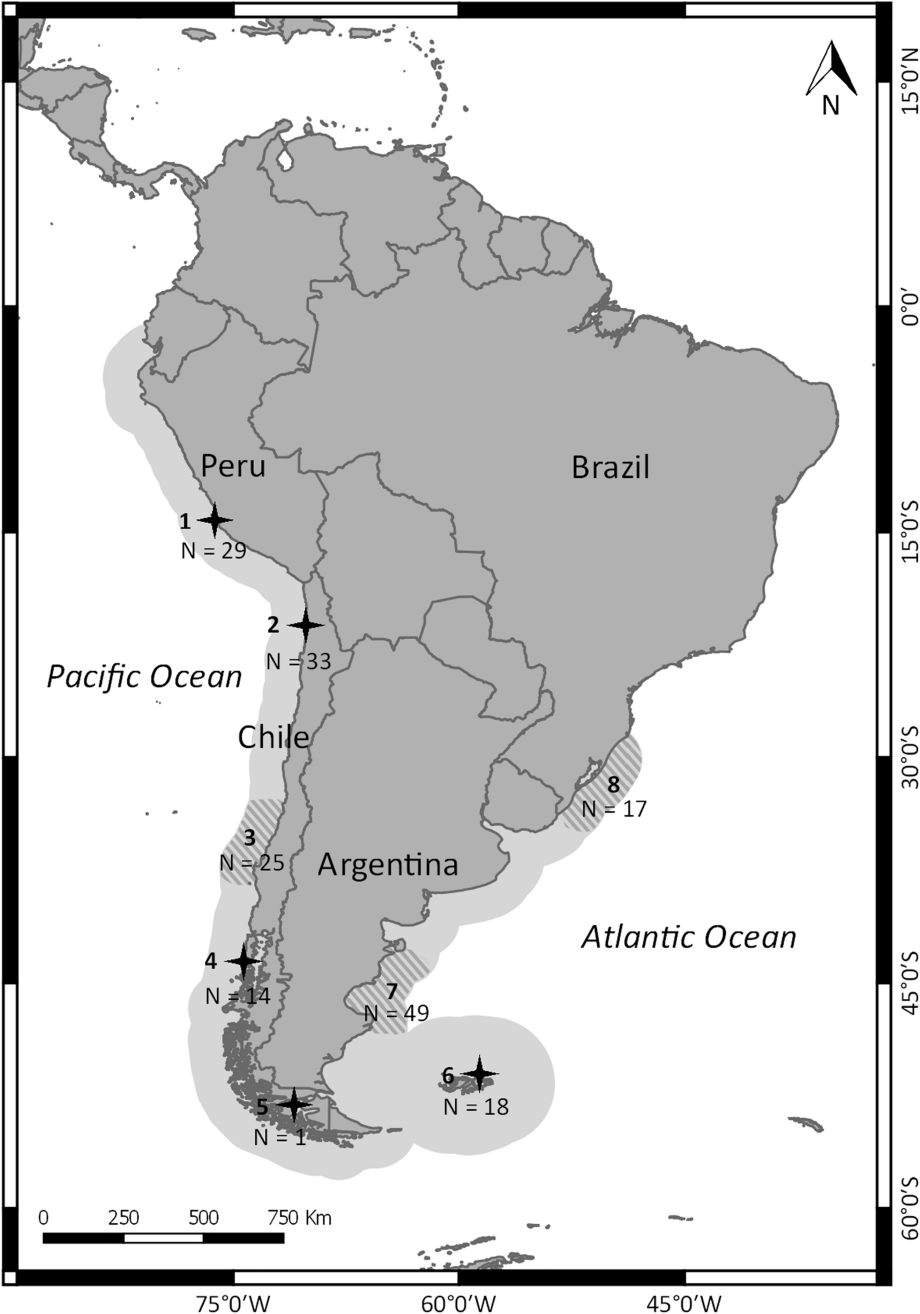
Sampling sites and geographic distribution of South American sea lion (light grey along the South American coast). Sampled localities were grouped in the following studied areas: Peru–Punta San Juan (1); Northern Chile—Punta Negra and Punta de Lobos (2); Southern Chile–between Ritoque beach and Isla Mocha (hatched lines 3), Guafo Island (4) and Punta Carrera (5); Falkland/Malvinas Islands (British Overseas Territory) (6); Argentina (hatched lines 7)–Argentine central coast; Uruguay (hatched lines 8)–southern Brazilian coast.

The South American sea lion *Otaria flavescens* (Shaw, 1800) (hereafter referred as sea lion) has a broad distribution in South America [7,8], covering approximately 10,000 Km of coast. In the Atlantic they are found from southern Brazil (29°S, 49°43'W) to Cape Horn, while in the Pacific they are found from Cape Horn to northern Peru (03°14'S, 80°34'W) [9] (Fig 1).

Skull morphometrics of *O*. *flavescens* show significant differences between the two sides of South America and support the existence at least of two [10] or even four populations of the species, two in the Atlantic and two in the Pacific [11]. Interestingly, these differences are more accentuated on the females than on the males [11]. The first genetic study of the species with allozymes did not show structure evidence between the colonies of Argentina and Uruguay within Atlantic coast [12]. However, most of the mitochondrial analyses performed to date are somewhat in line with the morphological results, showing strong and significant differentiation (high fixation index) with almost total absence of haplotype sharing between Pacific and Atlantic localities [8,13–16], although the most recent and extensive study that included sequences from Chile and Falkland Islands found two shared haplotypes between these areas [14]. These mtDNA differences have led some authors to propose two evolutionarily significant units (ESUs), one in each ocean [8,15,16]. Only the Atlantic Ocean presented data for molecular diversity between colonies using mtDNA and microsatellite loci with the matrilineal marker suggesting a significant difference between distant colonies [8,13–16]. However, the analysis of 13 microsatellite loci from 13 localities shows no significant difference and hence no population structure in the Atlantic [16]. These contrasting scenarios may be explained by philopatry of females and high dispersal of males [7], which is corroborated by a recent capture-recapture study [17], although this may not be valid for all areas, since biologging studies at Falkland/Malvinas Islands over winter showed that males did not undertake extended seasonal movements in this region [18]. A recent study with mtDNA from several areas across South America, including Falkland/Malvinas suggested a very strong mitochondrial structuring across the South American sea lion geographical range [14].

Philopatry of females with male-mediated gene flow is a common pattern in many species of pinnipeds [19,20,21]. Thus, the previous analyses of only matrilineal genes (mtDNA) might lead to misleading conclusions about the genetic structure of the species, and the presence of units important to conservation, such as ESUs and management units (MUs). An ESU should be reciprocally monophyletic for mitochondrial haplotypes and present significantly divergent at nuclear loci, while if only the differences in allele frequencies are present, it should be considered as a MU [22]. The identification of ESUs and MUS are very important for conservation planning because it allows the implementation of more effective strategies, where the genetic diversity of the species is considered [22].

Populations of sea lions suffered drastic reductions in the past two centuries [23]. The species has been hunted since pre-Columbian times with an increase in intensity after 1515 with the arrival of the explorer Juan Díaz de Solis [24]. The heaviest hunting period was in the first half of XX century, especially in the reproductive areas of the Atlantic coast [24–27]. Over the continent, sealing ceased mostly in the 1960’s and 1970’s (e.g. [18, 27]), although in Uruguay it remained until 1991 [24]. In Falkland/Malvinas Islands, the species suffered population declines between 1930 and 1965 [25,26], which is still poorly understood. High levels of mortality due to starvation were observed during *El Niño* events in Peru [28] as well as from strong interaction with the fishing activity along its entire distribution. The latest has been considered as the most important conservation problem faced by the species (for a review see [23]). Nevertheless, the sea lion has an estimated current census size of ~400,000 individuals (roughly 120,000 in Argentina, Peru and Chile; < 30,000 in Falkland/Malvinas Islands, and 12,000 in Uruguay [18, 23]) and is listed as Least Concern (LC) in the IUCN red list (www.iucnredlist.com). Furthermore, microsatellite data showed no association between genetic and demographic bottlenecks in Falkland/Malvinas Islands population [14] and, mismatch distribution analysis of mtDNA data suggests population expansion around ~45 thousand years ago (kya) for some Patagonians colonies [15], while the other suggested a population expansion around 25 ka for a single Patagonian area or for the whole Atlantic region [16].

Here we present the first genetic study on the South American sea lions that analyzed microsatellite and mtDNA data from the main areas of occurrence of the species in both the Atlantic and Pacific Oceans. We hypothesize that, besides a divergence between Atlantic and Pacific populations, the demographic history of the species would be influenced by Pleistocene climate cycles. In this context, we aim to: (1) characterize the genetic diversity and geographical structure across the species range; (2) test whether the previous population structure with two oceanic ESUs proposed using mtDNA are also supported by microsatellite data and estimate their divergence time; (3) investigate recent migration between populations; and (4) examine the demographic history of the species.

## Material and methods

### Ethics statement

All necessary permits were obtained to collect the samples for the described study, which complied with all relevant regulations. Samples from Brazil were collected with permit N° 020-04/CMA-IBAMA, issued by the current ‘Instituto Chico Mendes de Conservacão da Biodiversidade’; in Argentina permits issued by the ‘Dirección de Fauna y Flora Silvestre, Subsecretaria de Recursos Naturales, Ministerio de Industria, Agricultura y Ganaderia’; and by the ‘Subsecretaría de Conservación y Áreas Protegidas, Secretaría de Turismo’ permits N° 008/05, N° 178/07, N° 012/08, N° 003/09, N° 196/09, N° 001/10, N° 011/11, N° 082/12, N° 304/13 and N° 032/14. In Peru, samples were collected with permit N° 119-2006-INRENA-IFFS-DCB, issue by the “Instituto Nacional de Recursos Naturales”, in Chile issued by the current ‘Subsecretaría de Pesca” permits: N° 2100/2007, 786/2011, N° 2799/2008, N° 2396/2009, N° 1737/2010, N° 2794/2010. In Falkland/Malvinas Islands specimens were collected in 1991 and 1992 by researchers from the Sea Mammal Research Unit in affiliation with the British Antarctic Survey (BAS), working with the approval of the Falkland Islands Government, but no formal permits were required at that time. The sampling permits issued by local authorities in each country attest that the authors did not violate any ethical rule for collecting the animal samples. No samples were donated or purchased, and no sea lion was killed for the purpose of this study. All the samples used for this research derived from animals stranded and naturally dead, or from biopsied animals that were immediately released after the sampling activity.

### Field sampling and DNA extraction

A total of 150 *O*. *flavescens* tissue samples were collected from skin biopsies or from dead animals found ashore (Fig 1 and S1 and S2 Tables) between 1998 and 2011. In the last case, samples were collected from carcases of adult sea lions found along the Brazilian coast, specimens that probably came from Uruguay (see further explanation below [29, 30]). However, in the remaining sampling areas, the skin samples of ~0.5 cm^3^ were collected using piglet ear notch pliers from pups born at the colonies, with no anaesthetics due to the risk on pups [31]. All sampling equipment was sterilized with ethanol between uses. The samples were stored in ethanol 70% or DMSO [32]. Genomic DNA extractions were performed with standard phenol-chloroform [33] and NaCl protocols [34] or the DNeasy Tissue Kit (Qiagen).

Sampling sites were grouped in the following studied areas: (1) ‘**Peru’**: Punta San Juan (15°22’S; 75°11’W); (2) ‘**Northern Chile’**: between Punta Negra (20°09’S; 70°09’W) and Punta de Lobo (21°01’S; 70°10W); (3) ‘**Southern Chile’**: between Ritoque beach (32°49’S; 71°31’W) and Isla Mocha (38°20’S; 73°54’W), (4) Isla Guafo (43°33’S; 74°40’W), and (5) Punta Carrera (53°35’S; 70°55’W); (6) **Falkland/ Malvinas Islands (British Overseas Territory)**: Seal Bay (51°22'28" S; 58°02‴44 W’ and Stick in the Mud Islet (51°51'38" S; 61°10'05" W); (7) ‘**Argentina’**: between Punta Pirámide (42°35’S; 64°15W) and Isla Pinguino (47°54’S; 66°31W); and (8) ‘**Uruguay’**: from dead animals found ashore collected along southern Brazilian coast (32°17’S; 52°15’W). This latter arrangement was because, during the austral autumn and spring months, many sea lions are found along the Brazilian coast as a result of the dispersal of individuals from the closest Uruguayan natal colonies, such as Cabo Polonio and Isla de Lobos, after the breeding period [29, 30]. Samples from sites 3 to 5 were grouped as the Southern Chile study area since their individual sample sizes were small and exploratory analyses (not shown) suggested the populations were not statistically different.

### Mitochondrial DNA amplification, sequencing and analysis

The mitochondrial DNA control region (CR) and cytochrome *b* (cytb) gene were partially amplified by PCR using the following primers: L15926 5´- TCA AAG CTT ACA CCA GTC TTG TAA ACC—3´ [35]; H16498 5´- CCT GAA GTA GGA ACC AGA TG—3´ [36] for the control region and GLUDG-L 5′-TGA CTT GAA RAA CCA YCG TTG-3′ and CB2-H 5′- CCC TCA GAA TGA TAT TTG TCC TCA-3′ [37] for the cytb. Amplifications for CR and cytb were carried out in 20 μl with the following conditions: 1.5 mM MgCl2, 200 μM of each dNTP, 0.1 μM of each primer, 1 U of Platinum Taq DNA polymerase (Invitrogen), 1X PCR buffer (Invitrogen), 0.2%–0.4% Triton and 2 μl of DNA (approximately 50 ng). Thermocycling conditions were: 3 min at 94°C; 10 cycles of “touchdown”, each including 50 s at 94°C, 50s at 60°C (-1°C/cycle), and 80 s at 72°C; 30 cycles of 50 s at 94°C, 50 s at 50°C and 80 s at 72°C; followed by a final extension of 5 min at 72°C. Amplification products were purified with shrimp alkaline phosphatase and exonuclease I (Amersham Biosciences). The purified products were sequenced in both directions using the DYEnamic ET Dye Terminator Cycle Sequencing Kit (Amersham Biosciences) and run in a MegaBace 1000 automated sequencer (Amersham Biosciences). Few sequences from southern Chile were sequenced using an ABI 373A or an ABI 377 automated sequencer. The chromatograms were checked by eye in CHROMASPRO 1.7.4 (http://technelysium.com.au). Sequences were then aligned automatically within CLUSTALW 2.1 [38] and manually edited using BIOEDIT 7.1.3 [39].

Since we obtained mtDNA cytb sequences from only a few individuals from Chile, and this segment was not very informative at the population level, cytb sequences were used only in the network and phylogenetic inferences. Haplotype (Hd) and nucleotide diversities (π) were estimated using ARLEQUIN 3.5.1.2 [40]. Haplotype networks were constructed using the median-joining approach [41] implemented in NETWORK 4.6.11 (http://www.fluxus-engineering.com). Pairwise *F-*statistics (Φ_ST_ using p-distance) between populations and hierarchical Analysis of Molecular Variance (AMOVA) approach were estimated with ARLEQUIN using 10,000 permutations. Sequential Bonferroni correction was applied to the *P*-values to correct for multiple comparisons.

Divergence times between *O*. *flavescens* haplotypes were estimated using 103 cyt *b* sequences (Peru = 28, Southern Chile = 6, Uruguay = 14, Falkland/Malvinas Islands = 19, Argentina = 36) (see S2 Table). A fragment of 381 bp was aligned with 17 sequences of otariid species downloaded from GenBank. The sea lion species *Zalophus californianus*, *Z*. *wollebaeki* and *Eumetopias jubatus* were used as outgroup. We also included in the analysis one cyt *b* fragment sequence of eight species of *Arctocephalus*, *Neophoca cinerea* and *Phocarctos hookeri* (see S3 Table). A phylogenetic Bayesian approach was performed in BEAST 1.8.2 package. The parameters and priors used were a strict molecular clock, HKY substitution model with gamma site heterogeneity with six categories (model inferred by JMODELTEST2 [42,43]) and a Yule tree prior. To calibrate the phylogeny we used the divergence time between *Z*. *californianus*, *E*. *jubatus* and *O*. *flavescens* (6.7 ± 1.0 Ma), and between *O*. *flavescens* and the genus *Arctocephalus* (5.8 ± 1.0 Ma) [44], with a normal distribution. We also set a broad prior for the mean substitution rate using an estimate of 2%/site/Ma [45] (we used a lognormal distribution with standard deviation of one order of magnitude). We run two independent chains with 20,000,000 steps saved every 2,000 with a burn-in of 10%. The results were checked in TRACER 1.6 [46], summarized in TREEANOTATOR, and the phylogenetic tree drawn with FIGTREE 1.4.2 (http://tree.bio.ed.ac.uk/software/figtree/).

Demographic history for the geographic areas with a sample size larger than 25 were estimated using the Extended Bayesian skyline plot reconstructions on the mtDNA control region dataset performed with BEASTv2.4.3. The parameters and priors used were the Extended Bayesian Skyline Plot tree prior, HKY substitution model with gamma site heterogeneity with six categories, a strict clock rate was set to a lognormal distribution with a mean of 5.8% (standard deviation of 0.4) per million years (derived for *Callorhinus ursinus* [47]). We run chains with 100,000,000 steps, saved every 10000 and traces were examined for convergence in Tracer v.1.6 and used a burn-in of 10%. The skyline was calculated and plotted using the plotEBSP R script available at the BEAST web site assuming a generation time of 12 years [16].

### Microsatellite genotyping and analysis

We genotyped 111 samples for ten loci of dinucleotide short tandem repeats (STR): ZcwB07, ZcwE04, ZcwG04, ZcwF07 and ZcwE12 developed for *Z*. *californianus*; Hg8.10 and Hg6.3 developed for *Halichoerus grypus*; PvcE and Pv9 described for *Phoca vitulina*; and M11A described for *Mirounga* sp. [48,49,50,51]. Forward primers were 5’- tailed with the M13 sequence (5’- CACGACGTTGTAAAACGAC– 3’) that was used in combination with a M13 primer marked with fluorescence (FAM, HEX, NED) [52]. Amplifications were carried out in 10 μL with the following conditions: 1.5 mM MgCl2, 0.2 mM of each dNTP, 0.2 μM of reverse and M13-fluorescent primers, 0.133 μM of the M13-tailed forward primer, 0.5 U of Platinum Taq DNA polymerase (Invitrogen), 1X PCR buffer (Invitrogen), 0.6% of Trehalose and 2 μl of DNA (approximately 50 ng). Thermocycling conditions for the amplification of the loci were: 2 min at 94°C; 40 cycles of 45 s at 94°C, 45 s at annealing temperatures described in the original descriptions above, 50 s at 72°C; and a final extension of 2 min at 72°C. The PCR products were genotyped on a MegaBACE 1000 automated sequencer (Amersham Biosciences). The allele size in number of bases was identified with the software GENETIC PROFILER 2.2 (Amersham Biosciences) and the genotypes were normalized and binned using the program ALLELOGRAM. Micro-checker was used to test for the presence of genotype errors, evidence of null alleles, stuttering and allele dropout in populations [53].

Significant departure (α = 0.05) from Hardy-Weinberg equilibrium (HWE) was determined using ARLEQUIN exact test with 100,000 steps Markov chain and 1000 dememorization steps. Expected (He) and observed heterozygosity (Ho), pairwise differentiation (using the sum of squared differences, R_ST_-like method) between populations, and AMOVA were estimated with ARLEQUIN using 10,000 permutations. Sequential Bonferroni correction was applied to the *P*-values to correct for multiple comparisons.

Genetic population structure was assessed by the Bayesian approach implemented in the program STRUCTURE 2.3.4 [54]. First, we performed 10 independent runs for each *K* from *K* = 1 to *K* = 10, applying 1,000,000 Markov chain Monte Carlo (MCMC) iterations and a burn-in period of 1,000,000, using sampling locations (the six sampling areas defined above) as prior information to assist the clustering with the LOCPRIOR model [55]. The optimal number of genetic clusters was determined using the Evanno’s method [56] as implemented by STRUCTURE HARVESTER [57]. The existence of substructure within each ocean basin was tested (from K = 1 to 3) in separate runs as above. The multiple runs were merged and plotted using the online tool Pophelper 1.0.7 (http://pophelper.com). Finally, we tested for migrants or hybrids between oceans applying the USEPOPINFO model of the STRUCTURE software (options MIGPRIOR = 0.05 and GENSBACK = 0; the other run parameters were as above). This model uses pre-defined groups/clusters as priors (in this case ocean basins–see STRUCTURE results) to test for migrants or hybrids.

Contemporary gene flow was estimated using a Bayesian method implemented in BAYESASS 3.0.3 [58]. We estimated the migration rates (the proportion of individuals in a population that immigrated from a source population per generation) on two scenarios, one between the two oceans as a whole and the other between the six sampling areas. In the following description of the parameters, the first value refers to the former and the second to the latter scenarios. The analyses run for 3 x 10^7^/2 x 10^8^ steps that recorded every 1,000/2,000 iterations, with the first 3x10^6^/5 x 10^6^ discarded as burn-in. To reach the recommended acceptance rates between 20% and 60%, the values of parameters such as migration rates (Δ_M_), allele frequencies (Δ_A_) and inbreeding coefficient (Δ_F_) were adjusted to 0.1/0.9, 0.3/0.8 and 0.5/1, respectively. Trace files were examined for convergence to its stationary distribution in TRACER.

The genetic distance between pairs of sampling areas was inferred by estimation of the *D*_*SW*_ distance [59] and this matrix was used to estimate a neighbor-joining unrooted tree of the areas with POPULATIONS 1.2.32 [60]. Support for tree nodes was assessed by bootstrapping across loci (1,000 iterations) and the resulting tree was displayed with FIGTREE (http://tree.bio.ed.ac.uk/software/figtree/).

## Results

### Molecular characteristics

We analyzed 147 mtDNA control region sequences of sea lions sampled in almost the entire range of the species (Fig 1 and S1 Table). A total of 376 base pairs were analyzed for sequence variation that resulted in 46 haplotypes and 39 polymorphic sites and overall high haplotype diversity (Table 1).

**Table 1:** Mitochondrial genetic and microsatellites diversities in each locality: N number of individuals analyzed (averaged over loci for microsatellites); Hd, haplotype diversity; π, nucleotide diversity; H, number of haplotypes; K, average number of alleles; Ho, observed heterozygosity; He, expected heterozygosity.

	Control region				Microsatellites			
Population and Ocean basin	N	Hd (SD)	π (SD) %	H	N	K	Ho	He
Peru	28	0.86 (0.057)	0.80 (0.1)	14	29	7.7	0.72	0.77
Northern Chile	15	0.94 (0.045)	1.56 (0.2)	11	19	6.7	0.77	0.79
Southern Chile	30	0.83 (0.036)	0.48 (0.07)	8	15	6.5	0.69	0.79
**Overall Pacific**	**73**	**0.89 (0.024)**	**0.83 (0.09)**	**23**	**63**	**9.4**	**0.73**	**0.79**
Falkland/Malvinas Islands	18	0.87 (0.047)	0.74 (0.09)	7	13	6.4	0.72	0.77
Argentina	43	0.83 (0.032)	0.73 (0.09)	12	20	7.9	0.79	0.79
Uruguay	13	0.90 (0.054)	0.66 (0.06)	7	15	5.5	0.51	0.70
**Overall Atlantic**	**74**	**0.93 (0.014)**	**1.04 (0.08)**	**26**	**48**	**9.3**	**0.66**	**0.77**
**Overall**	**147**	**0.95 (0.009)**	**1.91 (0.07)**	**46**	**111**	**11.2**	**0.70**	**0.81**

All sampling populations have similar high values of haplotype diversity (≥0.83) but relatively low nucleotide diversities (from ~0.5% to ~0.8%), except the northern Chilean population, that had the highest diversities (~1.6%) (Table 1). The haplotype diversities for the Pacific and the Atlantic basins are also very similar, but the overall nucleotide diversity is lower in the Pacific basin, with the southern and northern Chile presenting the lowest and the highest diversities of all areas, respectively.

A total of 111 individuals were genotyped at 10 polymorphic microsatellite loci with 11% of missing data (Table 1). Using Micro-checker we did not find any evidence of allele dropout, in any locus. In some populations one or two loci showed some signal of null allele or stuttering, but since there was no consistency in these loci between populations, no locus was excluded from the analyses. After Bonferroni corrections, only three loci deviated from HWE but they were not consistent between populations (S4 Table in Appendix for details). Overall, only 6% of within populations locus pairs demonstrated significant linkage disequilibrium (*P* <0.05) (S4 Table in Appendix), but again these were not consistent, indicating low multilocus interactions [61]. Since the deviations were not consistent across most loci and populations, we did not exclude any loci from the analyses. The species as a whole presented high levels of genetic diversity for ten microsatellite loci (mean A = 11.2 alleles and He = 0.81) (Table 1). Within populations, the loci varied from moderately to highly polymorphic, with allelic richness ranging from 5.5 in the Uruguayan population to 7.9 in the Argentine population. Additionally, the genetic diversity in the nuclear loci was also similar in the Pacific and Atlantic groups of individuals, like in the mtDNA results. The expected heterozygosity and average number of alleles for the two oceanic areas were very similar (He ≥0.77 and K ~9), even though the Pacific area had more analyzed specimens.

### Population structure

**Fig 2:**
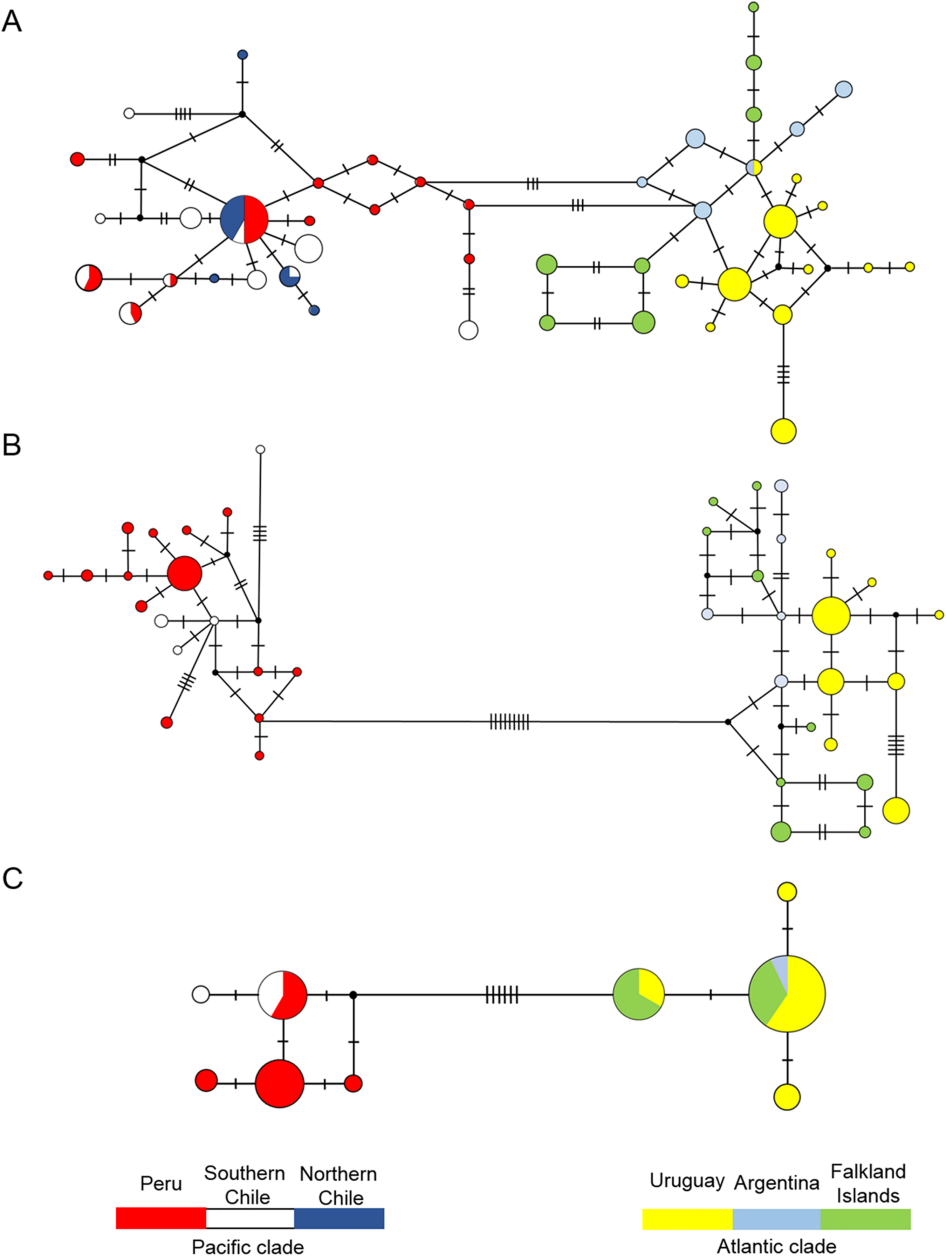
**Median-joining network of South American sea lion haplotypes based on the (A) control region sequences; (B) control region plus cyt b concatenated sequences; and (C) only cyt b sequences**. Each circle represents a unique haplotype, with size being proportional to the number of samples carrying it and cross lines the number of differences between then.

The mtDNA haplotype networks for the control region, cyt b, and the concatenated dataset showed two major and divergent clades corresponding to individuals that inhabit each ocean basin (Fig 2), that is, no haplotype is shared between individuals from Atlantic and Pacific, indicating high inter-oceanic matrilineal genetic structure. Haplotype sharing was much more frequent within Pacific than between Atlantic populations; in the latter there is a single shared control region haplotype between Uruguay and Argentina (Fig 2A), and in the control region plus cyt b results (Fig 2B) Argentina forms a monophyletic clade. With few exceptions, most haplotypes are separated by a single substitution.

**Fig 3:**
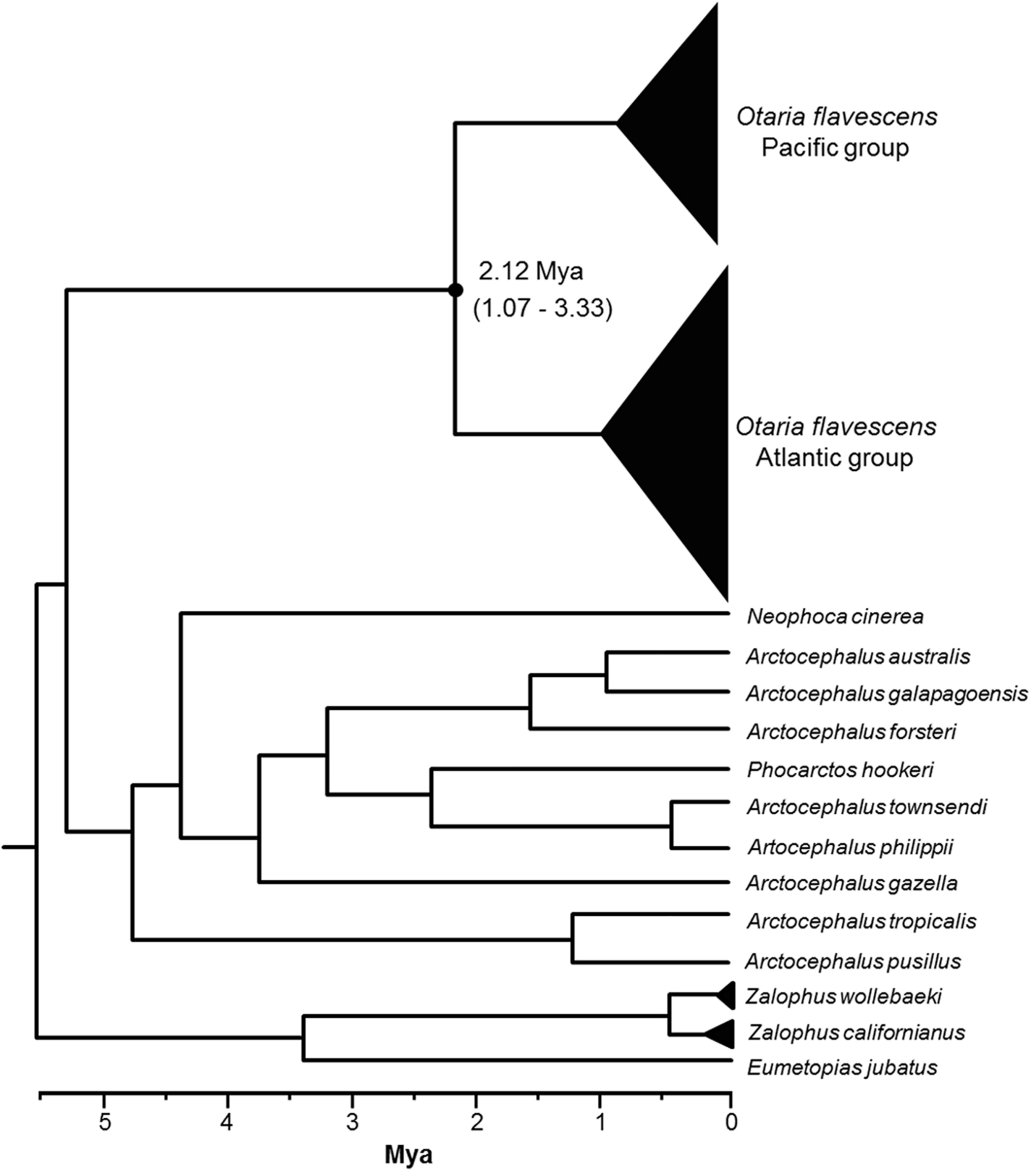
Bayesian phylogenetic tree of South American sea lion and related species *cyt b* sequences (381 bp). The two *Otaria flavescens* clades have a posterior probability of 1 and their median divergence time (in Mya) is indicated, with 95% credibility interval within parentheses.

In the Bayesian phylogenetic tree of the *cyt b* sequences, that includes sequences from most of the other species of the family, *Otaria* forms a monophyletic and basal clade (Fig 3). It also supports the presence of the Atlantic and Pacific clades with a high confidence (posterior probabilities of 1). The point estimate of the divergence time between these two clades, ~2 million years ago (Mya), is very large, being older than the estimated divergence time between several species in the family. The coalescence times within the Atlantic and Pacific clades are similar, around 1 Mya.

The mtDNA pairwise Φ_ST_ values between sampling areas were all high and significant, with the exception of the Peru versus northern Chile comparison (Table 2). Also, the pairwise difference between Atlantic and Pacific as a whole (Φst = 0.593; *P* < 0.001) was higher than any within-basins comparison, even between geographically closer populations from the southern tip of the continent but from different oceans, such as southern Chile versus Falkland/Malvinas Islands. The genetic distances between the three Atlantic populations were all larger than the distances between the three Pacific populations.

**Table 2:** Pairwise microsatellite R_ST_ (above diagonal) and mtDNA control region Φ_ST_ distance values (below diagonal) between the six sampling areas. Negative values were adjusted to zero.

Populations	Peru	Northern Chile	Southern Chile	Argentina	Falkland/ Malvinas Islands	Uruguay
Peru		0	0.019	0.070**	0.056*	0.187**
Northern Chile	0.038		0.034	0.061*	**0.071**	0.203**
Southern Chile	0.213**	0.170**		**0.020**	**0.022**	0.102*
Argentina	0.689**	0.598**	0.703**		0	0.029
Falkland/ Malvinas Islands	0.696**	0.625**	0.713**	0.456**		0.076
Uruguay	0.667**	0.556**	0.689**	0.384**	0.382**	

The significance is marked with *(*P* ≤ 0.05)

new significance after Bonferroni correction with **(*P* ≤ 0.003).

Values in bold are inter-oceanic comparisons.

**Fig 4:**
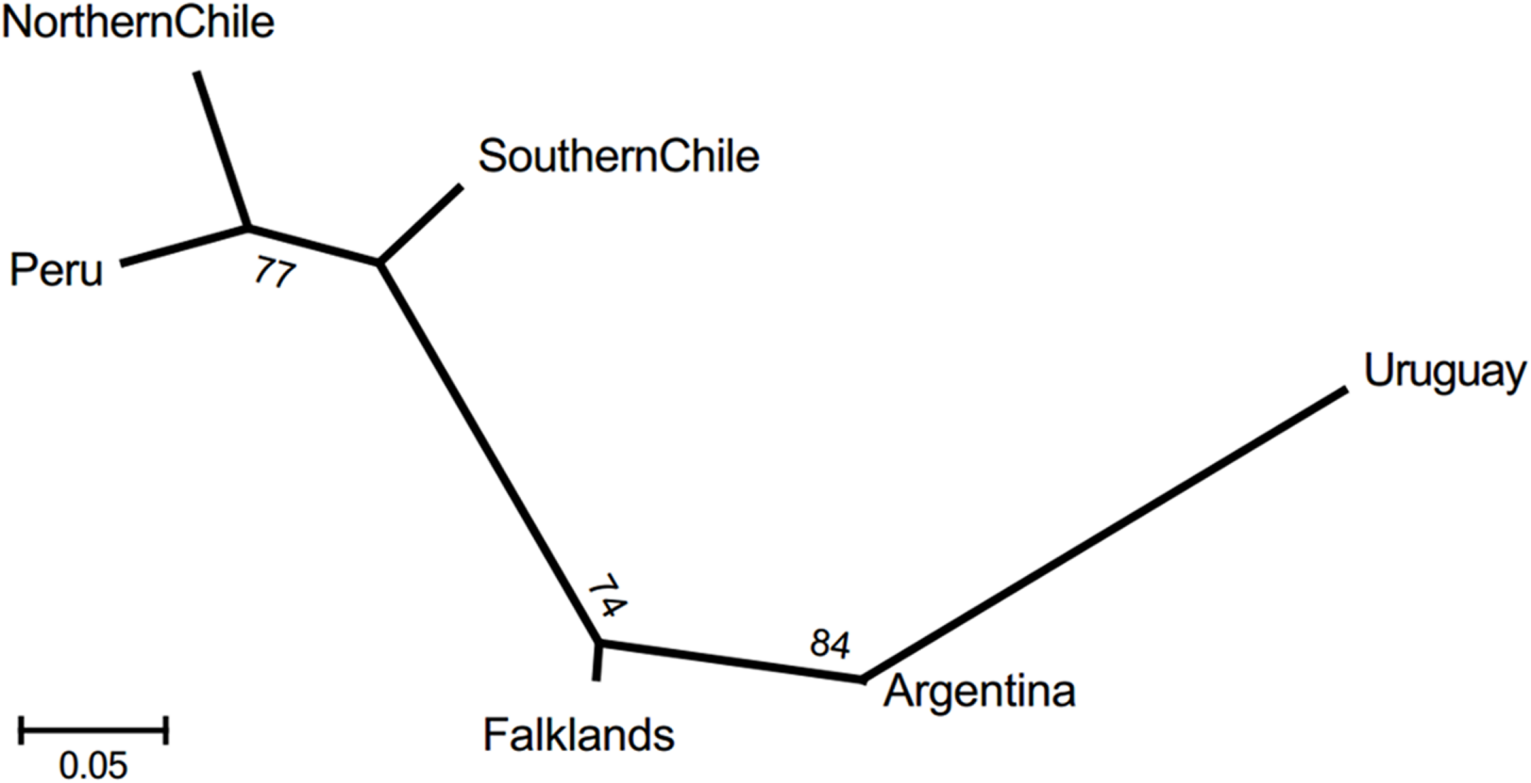
Unrooted neighbour-joining tree for South American sea lion populations using D_SW_ genetic distance on microsatellite data. Numbers are bootstrap values (%).

Microsatellite R_ST_ distance between Pacific and Atlantic was also significant (R_ST_ = 0.0892; *P* < 0.001). R_ST_ distances between the six sampling areas were not very high in general and all distances within-basins were not significant, as well as the distances between southern Chile and the Atlantic areas, with exception of the Uruguay (Table 2). Within each ocean basin, the lowest value was between the closest areas, Peru versus northern Chile, and Argentina versus Falkland/Malvinas Islands. The Uruguayan population is distinctively the most divergent. The neighbour-joining tree (Fig 4) derived from the microsatellite D_SW_ genetic distances, showed the large distance between Atlantic and Pacific sets of areas and that the Atlantic areas were more distant than the Pacific areas.

**Fig 5:**
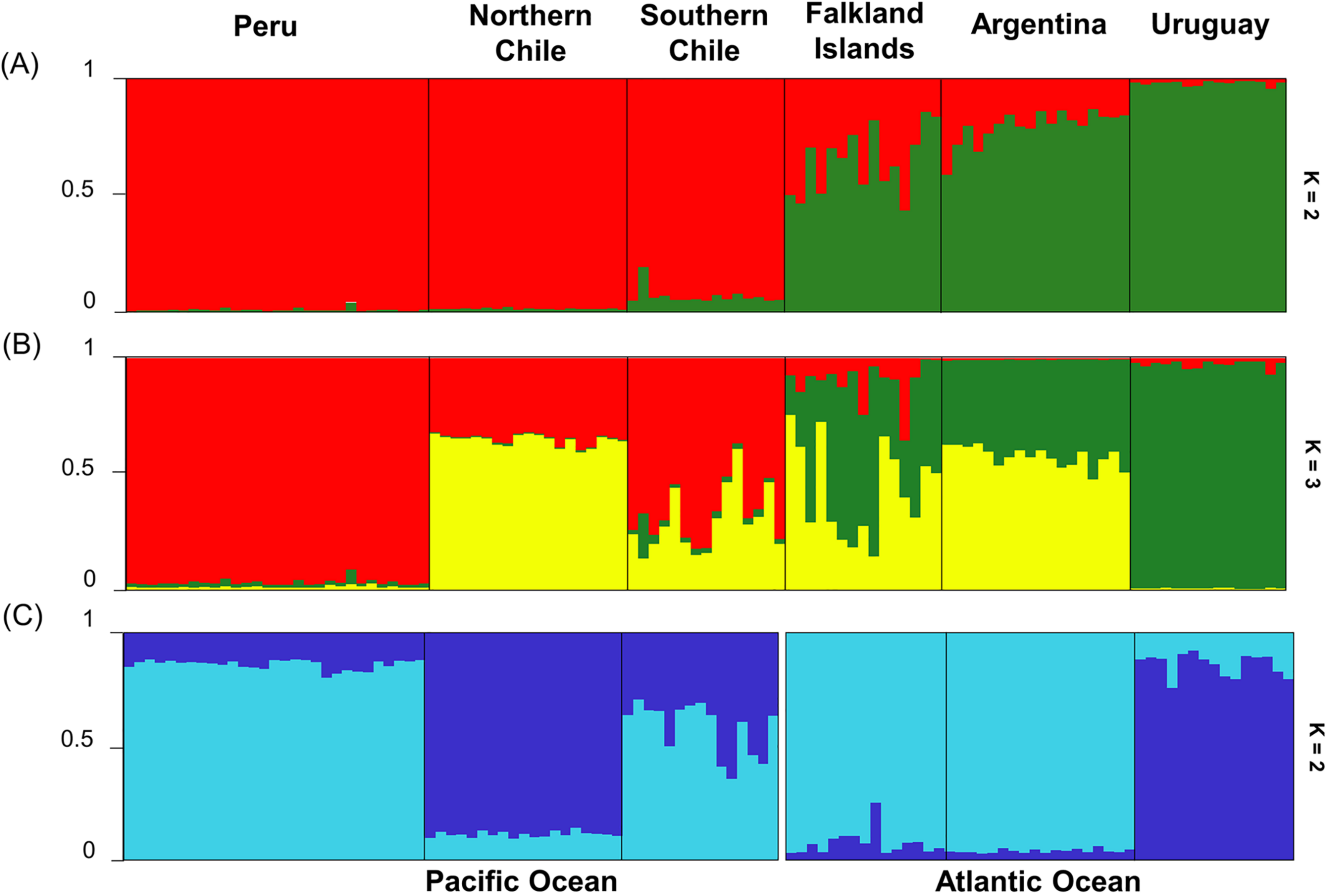
**STRUCTURE bar plots for South American sea lion populations considering (a) two and (b) three genetic clusters and (c) for each ocean separately.** Each bar is one individual and each colour represents the assignment probability of the individual to belong to that genetic cluster.

Results of STRUCTURE analysis of the microsatellite variation suggest the species is mainly structured in an Atlantic and a Pacific group (*K =* 2, Ln = -3598.96 by Evanno’s method) (Fig 5A, S1 Fig), although individuals from the Falkland/Malvinas Islands, and in lesser extent, Argentina have a reasonable proportion of membership in the cluster associated with the Pacific.

The test for migrants or hybrids between the two ocean basins performed in STRUCTURE suggested that only six individuals presented a proportion lower than 90% of the genetic component associated with their ocean of sampling (S2 Fig). Of these six, only two specimens, sampled in Atlantic (from Argentina and Falkland/ Malvinas Islands), presented a higher proportion (~70%) of the genetic component of the other ocean basin (Pacific). These individuals, especially the first three are likely first or second generation hybrids between oceans or, less likely, migrants as some individuals were adults. Two other individuals sampled in Falkland/Malvinas presented ~40% and 10% of the Pacific component. Two individuals, sampled in Peru and Southern Chile presented ~20% of the Atlantic component. The exploratory structure bar plot with *K* = 3 shown that, aside from the two northernmost populations from each ocean, Uruguay and Peru, the other populations seem to share a genetic component that is absent from the former two (Fig 5B), which may be related to some gene flow between them. The STRUCTURE analyses with the microsatellite data from each ocean separated detected significant substructure: two groups in the Atlantic, Uruguay and Argentina plus Falkland/Malvinas Islands and two components in the Pacific, with Peru and northern Chile with a high proportion of each one and, southern Chile a mixture of both components, but more similar to Peru (Fig 5C).

The BAYESASS analysis at the ocean level suggested an asymmetric and restricted contemporary gene flow between the two ocean basins, ~6.1% (standard error = 2.8%) from Pacific to Atlantic and ~1% per generation (standard error = 0.9%) from Atlantic to Pacific, although only the former is (marginally) significantly different from zero. The BAYESASS analysis considering the six areas presented mostly very low inter-oceanic migration rates (and all with confidence intervals that cross zero), although migration rates from some Pacific to some Atlantic areas were much higher than the others (northern Chile and Peru to Falkland/ Malvinas Islands and Argentina, Table 3). The migration rates between areas within each ocean are very asymmetric: they are much higher from North to South (ex. from Uruguay to Argentina and from Peru to southern and northern Chile) (although not all are significantly different from zero) while from South to North they are as small as the inter-oceanic migration rates (and all not significantly different from zero) (Table 3).

**Table 3:** Migration rates (%, standard error in parenthesis) between the six sampling areas along the South America based on microsatellite data. The migration rate is the proportion of individuals in a population that immigrated from a source population per generation. Values in bold are inter-oceanic comparisons.

To / From	Peru	Northern Chile	Southern Chile	Falkland/ Malvinas Islands	Argentina	Uruguay
Peru		2.67 (3.11)	1.06 (1.03)	**1.35 (1.27)**	**1.11 (1.07)**	**1.84 (1.56)**
Northern Chile	13.06 (10.08)		1.49 (1.44)	**1.75 (1.64)**	**1.95 (1.86)**	**1.43 (1.37)**
Southern Chile	19.76 (5.31)	6.84 (4.99)		**1.71 (1.63)**	**1.65 (1.57)**	**1.72 (1.64)**
Falkland/ Malvinas Islands	**6.94 (5.13)**	**6.69 (4.98)**	**1.83 (1.74)**		2.63 (2.68)	13.27 (4.31)
Argentina	**2.85 (2.52)**	**6.05 (4.66)**	**1.45 (1.41)**	1.77 (1.74)		19.46 (4.48)
Uruguay	**1.72 (1.64)**	**1.65 (1.57)**	**1.64 (1.56)**	1.72 (1.64)	1.91 (1.79)	

### Population history

**Fig 6:**
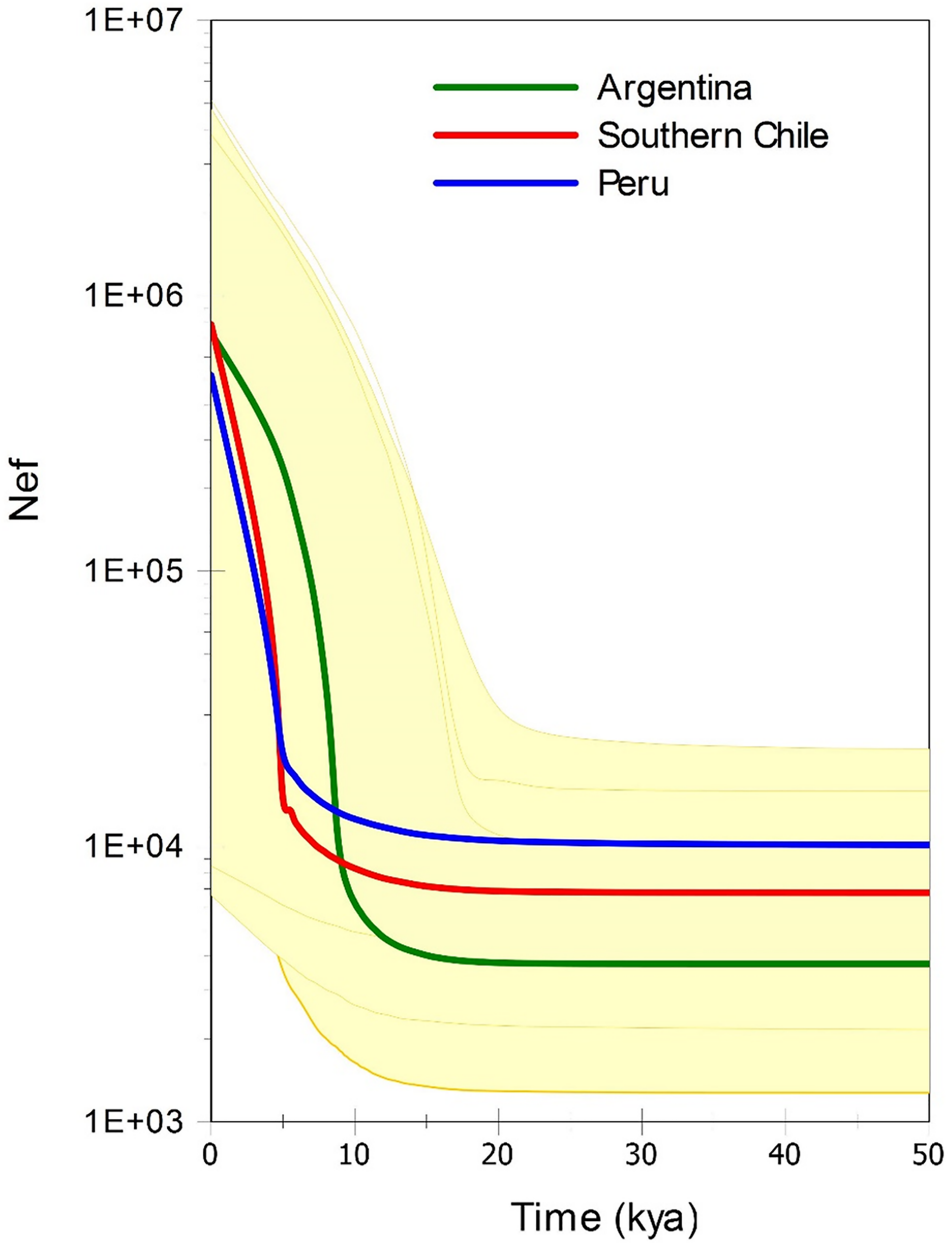
Extended Bayesian skyline plot showing the effective population size fluctuation of three South American sea lions populations throughout time based on the mtDNA control region. Internal thick lines are median estimates and thin lines and coloured areas are the 95% Central Posterior Density (CPD) intervals. Nef, effective female population size (log scale), ka, thousands of years ago. Time was truncated at 50 ka.

The Extended Bayesian Skyline Plots of the mtDNA control region sequences from Argentina, Peru, and southern Chile areas (Fig 6 and S3 Fig) are strikingly similar in their population size history. The median estimate for the three populations shows evidence for a sharp expansion of about two orders of magnitude over the past 10 kyr. These signals of population expansion are very consistent as can be seen in the plots of the individual population trajectories (S4 Fig).

## Discussion

### Ocean basins divergence

High levels of mtDNA divergence and significant differentiation in microsatellite loci were found between the Pacific and the Atlantic populations of the South American sea lions. Mitochondrial haplotypes of individuals sampled in the Atlantic and the Pacific form two very divergent (~2.1 Ma) and reciprocal monophyletic clades, even though we included individuals sampled from the southern tip of the continent, where the marine geographical distances between Atlantic and Pacific areas are very short (Fig 1). Our results are in agreement with previous reports, which found a deep divergence between the two haplotype groups in mtDNA [14,16], and major differentiation between ocean basins on skull morphology [11], as well as no cyt b shared haplotypes between populations of the two sides of the continent [8]. All results supported the Atlantic and Pacific areas as different ESUs (see below). This ancient divergence and complete reciprocal mtDNA monophyly, especially in sea lions, is even more striking considering the evidence of some males mediated gene flow between the oceans (see below) and therefore the absence of physical barriers to dispersal. A similar reciprocal monophyly between Atlantic and Pacific was found for the South American fur seal, *A*. *australis* [8,62], suggesting the existence of a clear and persistent barrier for female gene flow in both species. However, for both species, there is a gap of sampling in the southernmost tip of South America, a region where the gene flow between the Atlantic and Pacific populations potentially takes place. In other otariid seals, population structure was much weaker than suspected (or restrict to mtDNA genes), probably due to the species smaller spatial scale range and male mediate gene flow, such as in the Galápagos Islands for the local fur seal, *Arctocephalus galapagoensis* [21], and sea lion *Zalophus wollebaeki* [63].

The origin and long-term maintenance of this major phylogeographic break are probably related to Pleistocene climatic changes and the synergy of ecological barriers with high female philopatry. We could hypothesize that the ancient divergence of these two clades was the result of the first and perhaps only female dispersal of the South American sea lion from the Pacific to the Atlantic coast of South America, as suggested previously [64]. Since the only migration route between the two areas is at the southern tip of South America, the maintenance of this maternal break may be related to the harsh environmental conditions at this region during most of the time since the beginning of the Pleistocene glacial cycles. For example, during the Last Glacial Maximum (LGM) and until ~10 ka the Strait of Magellan is thought to have been glaciated [65] and at the freezing peaks of some glacial cycles the Antarctica ice may have expanded and reached the Beagle Channel [66,67]. Besides, Strait of Magellan was closed during most of the late Pleistocene when sea level reached below -35 m [68]. Therefore, it is likely that during most of the Pleistocene (with exception of the very brief interglacials such as the present one) the highly philopatric females of the sea lion populations of each side of South America were virtually isolated.

A question that emerges from these results is where the boundary between these clades is located (and therefore of the two ESUs), given the present absence of physical barriers between Atlantic and Pacific ESUs. A second question is how complete is this isolation. This boundary or contact zone should occur further south than our southernmost sampling areas at both ocean basins (about latitude 45°S; the southernmost site, since sampling size of site no. 5 is too small to be informative, Fig 1), since we found complete reciprocal monophyly. To answer these questions we would need an exhaustive sampling in the region, probably along the Strait of Magellan and/or in Tierra del Fuego channels, unfortunately, one of the most inaccessible parts of the species distribution. Indeed, the recent and extensive study on mtDNA [14] found one control region haplotype shared between Chile and Falkland/ Malvinas populations. The Chilean haplotype was found in a female from Punta Arenas in the Strait of Magellan [69], a region in contact with both the Pacific and the Atlantic oceans, suggesting this region is a zone of contact between the ESUs.

### Intra-oceanic structure and conservation implications

We found evidence for population structure within each ocean basin. However, similarly to the inter-oceanic differentiation, the magnitude of the population structure within each ocean basin widely differs between the maternal and biparental markers, probably caused by the strong sex-biased gene flow (see below). While all but one intra-oceanic mtDNA F_ST_ distances (Peru x northern Chile) were significant, six pairs of the equivalent microsatellite F_ST_ distances were significant (Table 2), suggesting some level of intra-oceanic structure. This pattern is agreement with previous studies on the genetic structure of the species in the Atlantic based on mtDNA [8,14,15] and both markers [14,16]. In the most extensive of previous studies on mtDNA [14], the highest migration rate was observed from Argentina to Brazil, and from Falkland/ Malvinas Islands to Brazil, both were attributed to proximity and prevailing currents [14]. Unfortunately, this study did not present microsatellite data for mainland colonies of the species, only for Falkland/ Malvinas Islands.

The mtDNA analysis showed that almost all breeding areas of the species seem well-differentiated, suggesting low or very low female gene flow between two ocean basins and supporting the existence of Pacific and Atlantic ESUs. On biparental markers (microsatellite), there seem to be two main genetic components in each ocean (Fig 5C), supporting the existence of two MUs within each ocean basin as well as two ESUs one in each side of South America. Taking into account both markers, the most differentiated populations were Uruguay and Peru. In the Atlantic, the northernmost population, Uruguay, seems differentiated from the southern populations, Argentina and Falkland/Malvinas Islands, as expected given the geographic distance (~1,930 km). Limited haplotype sharing between Falkland/Malvinas Islands and the South American mainland was previously reported (see Fig 3 in [14]), with two haplotypes in common between Falkland/ Malvinas and Chile, and one shared by Falkland/Malvinas and Argentina, suggesting some but very restricted maternal gene flow between Falkland/Malvinas Islands and South America [14]. Interestingly, in the Pacific, the two geographically closest areas, Peru and northern Chile (distant by ~750 km) presented different genetic components while the southern Chile area seems to be a mixture of both, although it is closer to Peru.

We also found that the genetic differentiation among the three Atlantic areas was higher than among the three Pacific areas both on the mtDNA and on the biparental markers, although in the later differentiation was much lower (Table 2 and Figs 2, 4 and 5). These differences in population structure between the two oceans may be the consequence of the more continuous distribution of the colonies in the Pacific than in the Atlantic [70], which could facilitate the gene flow between colonies of the Pacific. Moreover, the difference in the Atlantic areas may have been enhanced by the elimination of all breeding colonies of Buenos Aires Province during the sealing of XVIII, remaining only haul out sites of males (mainly in harbors) [71].

The genetic structure results are also in partial agreement with the comprehensive analysis of skull morphology of 331 females on this species, which found four geographic groups: two different populations on the Pacific (Southern Chile and Peru) and two in the Atlantic Ocean (Uruguay plus Northern Argentina, and Central plus Southern Argentina) [11]. However, our results do not agree with the suggestion that sea lions could be separated into two groups based on their skull morphology of 55 adult males [10,72]: one from Falkland/Malvinas Islands and the other comprising individuals from mainland South America (Chile, Peru, Argentina and Uruguay). This discrepancy between genetic and morphological results could be explained by the lumping strategy used the authors for the continental sample.

In conclusion, we suggest the existence of at least two management units (MU) in each oceanic ESU: the Atlantic group formed mainly by individuals from the Uruguay and southern populations (Argentina and Falkland/Malvinas Islands) and the Pacific group containing individuals from Peru and northern Chile (being the southern Chile a mixture of both) in the Pacific ESU. Both ESU and MU concepts have been widely used in conservation biology, the former being genetically highly differentiated (usually isolated) populations that have high priory for conservation, and the latter may be considered as populations whose degree of differentiation from others is sufficient so that they should be managed separately [73].

The existence of an Atlantic and a Pacific ESU for sea lions along the coast of South America (supported by genetic and morphological data) and also the different management Units (MU) identified in each ocean, are very important information for the management strategies of the species in each ocean basin, since they face different conservation problems in each area (*e*.*g*. population declines in the Pacific colonies due El Niño events [74]; fisheries interactions (including incidental capture) along the entire distribution of the species [23,75], and with salmon aquaculture in southern Chile [76], historical sealing along the Atlantic ocean [23,24] and even illegal poaching in Peru [77]. Moreover, the detection of a single main ESU along each entire ocean basin is particularly important for the implementation of an integrative management plan for all the countries that belong to the same ESU. Thus, both ESUs and MUs, should be managed differently in accordance with their respective conservation problems in each ocean basin, with the purpose to maintain their genetic singularity and integrity as reservoirs to the species as a whole.

It is important to highlight the results related to the Uruguayan population that showed low genetic variability (in both markers studied), and based on the microsatellite results it is the most divergent among the studied Atlantic populations. This genetic differentiation coupled with the conservation problems faced by the population from Uruguay, such as the long commercial sealing period [24], fishery interactions [78], and population decline due to unknown reasons [79], this population become an urgent priority, demanding more realistic management plans for its conservation.

However, geographically denser sampling and probably a higher number of informative markers are necessary to better define the limits between these units and to test the existence of additional ones.

### Philopatry and paternal gene flow

Our result of mtDNA showed reciprocal monophyly but reduced differentiation on biparental genetic markers between the ocean basins is consistent with a strong female philopatry and gene flow mainly mediated by males, a dispersal pattern commonly found in several pinniped species (e.g. [20,21,80]). This scenario is also supported by the pattern of population structure based on morphological differences between females and males [11].

The admixture analysis (S2 Fig) and the BAYESASS migration rates estimates suggest an asymmetric male gene flow between oceans, with a preferential Pacific to Atlantic direction. Interesting, although not mentioned by the authors, mtDNA migration rates also suggest a higher Pacific (Chile) to Atlantic migration rate (Fig 4 in [14]). A possible explanation for this asymmetry is the presence of the Antarctic Circumpolar Current, that flows clockwise from west to east around Antarctica and that is very close to the southern Chile coast at the Drake Passage [81]. On the other hand, another pattern that is suggested by the BAYESASS migration rates is a higher North to South gene flow within each ocean basin (Table 3). Their dispersal movements during El Niño events could explain this result, since in this period, South American fur seals and sea lions usually follow the anchoveta shoals (*Engraulis rigens*) to the south, where the sea surface temperature is colder than Peruvian waters [82]. Consequently, Chilean colonies of fur seals and sea lions increase in numbers like in 1997, when the northern local population of sea lions had mainly young animals (1–3 years) that probably migrated from Peru [83]. This affected principally Punta Patache, where an increase of 160% of females and young was observed in summer 1997/98 [83].

Moreover, this direction of migration is mainly counter-current, especially on the Pacific coast, where the Peru (or Humboldt) current flows northward [44]. However, there is no evidence of a dependence between sea lions and marine currents, indeed a recent study showed that males from northern Argentina could make long-distance round-trips (up to 700 km) from nonbreeding areas in northern Argentina to breeding sites in Patagonia and Uruguay, that are southward and northward respectively [17]. Therefore, more analyses are necessary to address the degree of asymmetry in the dispersal and gene flow between colonies and the potential factors, such as oceanic currents, that can explain the patterns we found.

### Historical demographic patterns

The pattern of long-term population size dynamics of Argentina, and for the first time of two Pacific populations, southern Chile and Peru, suggested the three populations underwent a two order of magnitude increase in the last 10 kya, more pronounced in the Argentina area (Figs 6, S3 and S4). Signals of population expansion for some Atlantic populations of sea lions were found previously [15,16] using the mismatch distribution method. However, expansion times found in those studies are older than those observed here (~45 ka [15], ~25 ka [16]), with exception of one estimate from North Patagonia of ~10 ka in the former study. The younger expansion times found should not be expected at first, since most of the areas sampled were the same among these studies (and several haplotypes were identical between the studies) and we used a faster substitution rate. However, the mismatch distribution approach used previously is a much more limited method for the estimation of population expansion than the EBSP used here (see [84]), only given accurate estimates on very specific conditions (see [85]) not tested in those studies. We therefore consider that our results better reflect the demographic history of sea lion populations.

The past demographic changes shown in Fig 6 are very similar to the dynamics of the temperature and sea level changes at the end of the last glacial cycle at the Patagonia and Antarctica [86]. Climatic changes at the end of the Pleistocene and the Holocene were proposed previously as an important factor for the population size changes of Atlantic populations of sea lions [15] and fur seals [87]. Although for the fur seals the estimated expansion time was older than those estimated here, it was suggested the expansion in the Atlantic occurred since the LGM, induced first by colonization of the exposed continental shelf. On the other hand, our results are more compatible with the hypothesis that population reduction occurred toward the end of the last glacial cycle, especially during the LGM, followed by expansions after the Last Glacial Termination, in both oceans.

Fig 6. Extended Bayesian skyline plot showing the effective population size fluctuation of three South American sea lions populations throughout time based on the mtDNA control region. Internal thick lines are median estimates and thin lines and coloured areas are the 95% Central Posterior Density (CPD) intervals. Nef, effective female population size (log scale), ka, thousands of years ago. Time was truncated at 50 ka.

It was suggested that this fluctuation was caused by habitat contraction during LGM, with the northern areas acting as refugia, followed by southward expansion after deglaciation [15]. This scenario seems more easily applied to the Pacific, where during the LGM shoreline habitats were covered by the Patagonian ice sheet from the southern tip of the continent to ~40°S [88] and therefore were likely uninhabitable by coastal breeding animals. In the Atlantic on the contrary, the consequence of the LGM was the exposure of the very large continental shelf, without the presence of ice [68]. However, our results do not support this northern refugia scenario, since we found no clear trend of higher genetic diversity in northern areas (Table 1) [89,90], and no difference in demographic histories between Peru and southern Chile (Fig 6). Beyond these large changes in coastal habitats, the end of the glacial cycle also altered marine productivity [91] and therefore the abundance of top predators, like sea lions. For example, marine productivity at the southern end of South America may have been reduced at the end the last glacial cycle until the onset of the Holocene by the large melt water fluxes to both Pacific and Atlantic from the adjacent Patagonian Ice-sheet during deglaciation [89,92], until reached presented day conditions.

## Supporting information

S1 Table List of individuals that bear each mitochondrial DNA control region haplotype, and the respective GenBank number. Absolute frequency in the sample and geographic distribution of haplotypes for South American sea lion.
(DOCX)

S2 Table List of individuals that bear each mitochondrial DNA cytochrome b haplotype. Absolute frequency in the sample and geographic distribution of haplotypes for South American sea lion.
(DOCX)

S3 Table Species and access number of sequences downloaded from GenBank used to estimate the Bayesian phylogeny (Fig 3). (DOCX)

S4 Table Genetic diversity of South American sea lions for each *locus* per clustered localities and for the species as whole. (A) Number of alleles, (E) = exclusive alleles, (Ho) observed heterozygosity, (He) expected heterozygosity.* Loci that deviated from HW equilibrium after Bonferroni correction.
(DOCX)

S1 Fig Plots from STRUCTURE HARVESTER performed with Evanno’s method. (A) Highest value of (ΔK) = 79.20 on K = 2. (B) Mean of estimated Ln probability of data (± sd) averaging ten runs from K = 1 to K = 10.
(TIF)

S2 Fig STRUCTURE bar plot from the test for migrants or hybrids between oceans using the sampling locations (in this case the ocean basin) and the USEPOPINFO model. Each bar is one individual and each colour represents the assignment probability of the individual to belong to that genetic cluster.
(TIF)

S3 Fig Extended Bayesian skyline plot showing the effective population size fluctuation of South American sea lions populations throughout time based on the mtDNA control region. Internal dashed lines are median estimates and thin lines and coloured areas are the 95% Central Posterior Density (CPD) intervals. Nef, effective female population size (log scale), kya, thousands of years ago.
(TIF)

S4 Fig Extended Bayesian skyline plot showing the effective population size fluctuation of South American sea lions populations throughout time based on the mtDNA control region. Internal black dashed lines are median estimates and thin lines are the 95% CPD intervals. Thin green lines are the individual population trajectories. Nef, effective female population size (log scale), kya, thousands of years ago.
(TIF)
