# Supplementary material for: Ancient female philopatry, asymmetric male gene flow, and synchronous population expansion support the influence of climatic oscillations on the evolution of South American sea lion (Otaria flavescens)
Source: PLoS One. 2017 Jun 27;12(6):e0179442. doi: 10.1371/journal.pone.0179442 (PMC5487037; doi:10.1371/journal.pone.0179442)
Supplement: S1 Table — Absolute frequency in the sample and geographic distribution of haplotypes for South American sea lion. (DOCX) [file pone.0179442.s001.docx]

*Plos One*

**SUPPORTING INFORMATION**

**Ancient female philopatry, asymmetric male gene flow, and synchronous population expansion support the influence of climatic oscillations on the evolution of** **South American sea lion (*Otaria flavescens*)**

Larissa Rosa de Oliveira, Marcelo C. M. Gehara, Lúcia D. Fraga, Fernando Lopes, Juan Ignacio Túnez, Marcelo H. Cassini, Patricia Majluf, Susana Cárdenas-Alayza, Héctor J. Pavés, Enrique Alberto Crespo, Nestor García, Rocío Loizaga de Castro, Rus Hoelzel, Maritza Sepúlveda, Carlos Olavarría, Victor Hugo Valiati, Renato Quiñones, Maria Jose Pérez-Alvarez, Paulo Henrique Ott and Sandro L. Bonatto

**S1 Table**. List of individuals that bear each mitochondrial DNA control region haplotype, and the respective GenBank number. Absolute frequency in the sample and geographic distribution of haplotypes for South American sea lion. Samples were collected between 1998 and 2011.

| **Haplotype** | **Individuals** | **Frequency** | **Locality** | **Country** | **GenBank accession number** |  |
| --- | --- | --- | --- | --- | --- | --- |
| Of01 | PSJ01, PSJ03, PSJ04, PSJ05, PSJ06, PSJ16, PSJ17, PSJ19, PSJ20, PSJ21, PSJ22, PSJ23, NC1,NC2, SC1, SC2, SC3, SC4, SC5, SC6, SC7, SC8, IG05, IG06 | 24 | Punta San Juan  Punta Negra  Punta de Lobo  Carranza,  Cobquecura,  Santa Maria,  Isla Mocha,  La Sebastiana,  Isla Guafo  Punta Carrera | Peru  Chile | KY859197 | |
| Of02 | PSJ02 | 1 | Punta San Juan | Peru | KY859198 | |
| Of03 | PSJ07, NC3 | 2 | Punta San Juan  Punta de Lobo | Peru  Chile | KY859199 | |
| Of04 | PSJ08 | 1 | Punta San Juan | Peru | KY859200 | |
| Of05 | PSJ09 | 1 | Punta San Juan | Peru | KY859201 | |
| Of06 | PSJ10 | 1 | Punta San Juan | Peru | KY859202 | |
| Of7 | PSJ13, PSJ15, PSJ28, PSJ29, NC4, NC5, NC6 | 7 | Punta San Juan  Playa Chauca  Punta Negra | Peru  Chile | KY859203 | |
| Of08 | PSJ14, PSJ25, NC7, NC8, NC9 | 5 | Punta San Juan  Punta Patache  Punta Negra  Punta de Lobo | Peru  Chile | KY859204 | |
| Of09 | PSJ18, PSJ26 | 2 | Punta San Juan | Peru | KY859205 | |
| Of10 | PSJ24 | 1 | Punta San Juan | Peru | KY859206 | |
| Of11 | PSJ27 | 1 | Punta San Juan | Peru | KY859207 | |
| Of12 | PSJ30 | 1 | Punta San Juan | Peru | KY859208 | |
| Of13 | NC10, NC11, NC12 | 3 | Playa Chauca  Punta Negra  Punta de Lobo | Chile | KY859209 | |
| Of14 | NC13 | 1 | Punta Negra | Chile | KY859210 | |
| Of15 | NC14, SC11, SC12, IG03 | 4 | Pabellón de Pica  Santa Maria  La Sebastiana  Isla Guafo | Chile | KY859211 | |
| Of16 | NC15 | 1 | Punta de Lobo | Chile | KY859212 | |
| Of17 | SC9, SC10, SC11, SC12, IG01 | 5 | Carranza  Cobquecura  La Sebastiana  Isla Guafo | Chile | KY859213 | |
| Of18 | SC13, SC14, SC15, SC16, SC17, IG07, IG08, IG10 | 8 | Cobquecura  Isla Mocha  La Sebastiana  Isla Guafo | Chile | KY859214 | |
| Of19 | SC18, SC19, SC20, SC21 | 4 | Cobquecura  Santa Maria  La Sebastiana | Chile | KY859215 | |
| Of20 | SC22 | 1 | Santa Maria | Chile | KY859216 | |
| Of21 | SC23 | 1 | Isla Mocha | Chile | KY859217 | |
| Of22 | IG04 | 1 | Isla Guafo | Chile | KY859218 | |
| Of23 | GEMARS516, GEMARS1189, Gordo | 3 | Rio Grande do Sul | Brazil | KY859219 | |
| Of24 | GEMARS553, IP7 | 2 | Rio Grande do Sul  Isla Pinguino | Brazil  Argentina | KY859220 | |
| Of25 | GEMARS554, GEMARS967, GEMARS1178 | 3 | Rio Grande do Sul | Brazil | KY859221 | |
| Of26 | GEMARS658, GEMARS667 | 2 | Rio Grande do Sul | Brazil | KY859222 | |
| Of27 | GEMARS809, GEMARS812, GEMARS992 | 3 | Rio Grande do Sul | Brazil | KY859223 | |
| Of28 | GEMARS822 | 1 | Rio Grande do Sul | Brazil | KY859224 | |
| Of29 | IVO1 | 1 | Isla Vernacci | Argentina | KY859225 | |
| Of30 | IVO2, IVO4, IVO5, ML20, ML28, ML31, ML42, ML49, ML52, ML53, ML54, IP4 | 12 | Isla Vernacci, Monte Loayza  Isla Pinguino | Argentina | KY859226 | |
| Of31 | IVO3, ML39, PP6, PP7 | 4 | Isla Vernacci  Monte Loayza  Punta Pirámide | Argentina | KY859227 | |
| Of32 | IVO7, ML7, ML40, ML41, ML55, IP1, IP5 | 7 | Isla Vernacci  Monte Loayza  Isla Pinguino | Argentina | KY859228 | |
| Of33 | IVO10 | 1 | Isla Vernacci | Argentina | KY859229 | |
| Of34 | ML2, ML3, ML14, ML19, ML21, ML22, ML25, ML35, ML51, PP5, IP3 | 11 | Monte Loayza  Punta Pirámide  Isla Pinguino |  | KY859230 | |
| Of35 | ML16 | 1 | Monte Loayza | Argentina | KY859231 | |
| Of36 | ML24, ML50 | 2 | Monte Loayza | Argentina | KY859232 | |
| Of37 | ML57 | 1 | Monte Loayza | Argentina | KY859233 | |
| Of38 | IP2 | 1 | Isla Pinguino | Argentina | KY859234 | |
| Of39 | IP6 | 1 | Isla Pinguino | Argentina | KY859235 | |
| Of40 | I9, I102, J4, A2, A6 | 5 | Falkland/Malvinas Islands | British Overseas Territory | KY859236 | |
| Of41 | J1, J3, J5, J6 | 4 | Falkland/Malvinas Islands | British Overseas Territory | KY859237 | |
| Of42 | J2 | 1 | Falkland/Malvinas Islands | British Overseas Territory | KY859238 | |
| Of43 | J7, J8 | 2 | Falkland/Malvinas Islands | British Overseas Territory | KY859239 | |
| Of44 | J9, J10 | 2 | Falkland/Malvinas Islands | British Overseas Territory | KY859240 | |
| Of45 | A1, A5 | 2 | Falkland/Malvinas Islands | British Overseas Territory | KY859241 | |
| Of46 | A3, A4 | 2 | Falkland/Malvinas Islands | British Overseas Territory | KY859242 | |
