# Supplementary material for: Ancient female philopatry, asymmetric male gene flow, and synchronous population expansion support the influence of climatic oscillations on the evolution of South American sea lion (Otaria flavescens)
Source: PLoS One. 2017 Jun 27;12(6):e0179442. doi: 10.1371/journal.pone.0179442 (PMC5487037; doi:10.1371/journal.pone.0179442)
Supplement: S2 Table — Absolute frequency in the sample and geographic distribution of haplotypes for South American sea lion. (DOCX) [file pone.0179442.s002.docx]

**S2 Table**. List of individuals that bear each mitochondrial DNA cytochrome b haplotype. Absolute frequency in the sample and geographic distribution of haplotypes for South American sea lion.

| **Haplotype** | **Individuals** | **Frequency** | **Locality** | **Country** | **GenBank accession number** |
| --- | --- | --- | --- | --- | --- |
| Of1 | PSJ01 PSJ02 PSJ03 PSJ04  PSJ05 PSJ06 PSJ07 PSJ09 PSJ13 PSJ14 PSJ15 PSJ16  PSJ19 PSJ20 PSJ22 PSJ23  PSJ25 PSJ29 | 18 | Punta San Juan | Peru | KY859243 |
| Of2 | PSJ17 PSJ21 | 2 | Punta San Juan | Peru | KY859244 |
| Of3 | PSJ24 | 1 | Punta San Juan | Peru | KY859245 |
| Of4 | IG04 IG05 IG07 IG08 IG10  PSJ08 PSJ10 PSJ11 PSJ18 PSJ26  PSJ27 PSJ30 | 12 | Isla Guafo Punta San Juan | Peru Chile | KY859246 |
| Of5 | IG06 | 1 | Isla Guafo | Chile | KY859247 |
| Of6 | G517 G553 G554 G555 G667 G809 G812 G813  G868 G967 G992 G1178 G1189 Gordo IVO2 IVO3 IVO6 IVO8 IVO10 PP5 PP6 PP7 ML2 ML8 ML10  ML14 ML16 ML20 ML21  ML28 ML35 ML39 ML42 ML49 ML51 ML53 ML54 ML57 IP4 FKL4 FKL9 FKL10 | 42 | Rio Grande do Sul Isla Vernacci Punta Pirámide Monte Loayza Falkland/Malvinas Islands | Brasil Argentina British Overseas Territory | KY859248 |
| Of7 | IVO7 ML1 ML7 ML40 ML41  IP1 IP5 FKL1 FKL2 FKL3 FKL5 FKL6 FKL7 FKL8 FKL11 FKL12 FKL13  FKL14 FKL15 FKL18 FKL19 | 21 | Isla Vernacci Monte Loayza Isla Pinguino Falkland/Malvinas Island | Argentina  British Overseas Territory | KY859249 |
| Of8 | ML6 ML22 ML25 IP3 | 4 | Monte Loayza Isla Pinguino | Argentina | KY859250 |
| Of9 | FKL16 FKL17 | 2 | Falkland/Malvinas Island | British Overseas Territory | KY859251 |
