# Supplementary material for: Ancient female philopatry, asymmetric male gene flow, and synchronous population expansion support the influence of climatic oscillations on the evolution of South American sea lion (Otaria flavescens)
Source: PLoS One. 2017 Jun 27;12(6):e0179442. doi: 10.1371/journal.pone.0179442 (PMC5487037; doi:10.1371/journal.pone.0179442)
Supplement: S3 Table — (DOCX) [file pone.0179442.s003.docx]

**S3 Table**. Species and access number of sequences downloaded from GenBank used to estimate the Bayesian phylogeny (Fig 3).

| **Species** | **GenBank accession number** |
| --- | --- |
| *Zalophus californianus* | AM422164, AM422163, AM422156 |
| *Zalophus wollebaeki* | AM422150, AM422151, AM422152 |
| *Eumetopias jubatus* | FJ948490 |
| *Arctocephalus tropicalis* | AF380886 |
| *Arctocephalus pusillus* | AF380916 |
| *Arctocephalus townsendi* | AF 380897 |
| *Arctocephalus philippii* | AF380896 |
| *Arctocephalus gazella* | AF380882 |
| *Arctocephalus australis* | AY712969 |
| *Arctocephalus forsteri* | JN588761 |
| *Arctocephalus galapagoensis* | AF380900 |
| *Neophoca cinerea* | AF380915 |
| *Phocarctos hookeri* | AF380919 |
