# Supplementary material for: Ancient female philopatry, asymmetric male gene flow, and synchronous population expansion support the influence of climatic oscillations on the evolution of South American sea lion (Otaria flavescens)
Source: PLoS One. 2017 Jun 27;12(6):e0179442. doi: 10.1371/journal.pone.0179442 (PMC5487037; doi:10.1371/journal.pone.0179442)
Supplement: S4 Table — (A) Number of alleles, (E) = exclusive alleles, (Ho) observed heterozygosity, (He) expected heterozygosity.* Loci that deviated from HW equilibrium after Bonferroni correction. (DOCX) [file pone.0179442.s004.docx]

**S4 Table**. Genetic diversity of South American sea lions for each *locus* per clustered localities and for the species as whole. (A) Number of alleles, (E) = exclusive alleles, (Ho) observed heterozygosity, (He) expected heterozygosity.* Loci that deviated from HW equilibrium after Bonferroni correction.

| **Locus** | **Allele size interval (bp)** | **Peru** | | | |  |  | **Northern Chile** | | | | |  | **Southern Chile** | | | | | | **Falkland Islands** | | | |  |
| --- | --- | --- | --- | --- | --- | --- | --- | --- | --- | --- | --- | --- | --- | --- | --- | --- | --- | --- | --- | --- | --- | --- | --- | --- |
|  |  | **A** | **E** | **Ho** | **He** |  | **A** | **E** | **Ho** | **He** |  | **A** | | | **E** | **Ho** | **He** |  | **A** | | **E** | **Ho** | **He** |  |
| ZcwB07 | 192-212 | 6 | 0 | 0.83 | 0.81 |  | 6 | 0 | 0.63 | 0.81 |  | 7 | | | 1 | 0.60 | 0.81 |  | 6 | | 0 | 0.78 | 0.80 |  |
| Hg8.10 | 200-238 | 13 | 2 | 0.79 | 0.89 |  | 9 | 0 | 0.65 | 0.88 |  | 8 | | | 0 | 0.82 | 0.87 |  | 9 | | 0 | 0.80 | 0.90 |  |
| ZcwE04 | 125-141 | 5 | 2 | 0.62 | 0.54 |  | 3 | 0 | 0.88 | 0.63 |  | 4 | | | 0 | 0.53 | 0.63 |  | 2 | | 0 | 0.00 | 0.19 |  |
| PvcE* | 128-146 | 7 | 0 | 0.28 | 0.71 |  | 8 | 1 | 0.47 | 0.84 |  | 7 | | | 1 | 0.57 | 0.80 |  | 6 | | 0 | 1.00 | 0.78 |  |
| M11A | 173-197 | 7 | 1 | 0.79 | 0.79 |  | 7 | 0 | 0.74 | 0.78 |  | 5 | | | 0 | 0.70 | 0.76 |  | 10 | | 0 | 0.80 | 0.86 |  |
| ZcwG04 | 172-212 | 9 | 1 | 0.93 | 0.86 |  | 9 | 2 | 0.79 | 089 |  | 7 | | | 0 | 0.67 | 0.80 |  | 7 | | 0 | 0.70 | 0.87 |  |
| Pv9 | 193-213 | 8 | 1 | 0.90 | 0.85 |  | 7 | 0 | 1.00 | 0.87 |  | 5 | | | 0 | 0.80 | 0.83 |  | 5 | | 0 | 1.00 | 0.84 |  |
| Hg6.3* | 242-258 | 5 | 0 | 0.41 | 0.62 |  | 5 | 0 | 0.81 | 0.70 |  | 6 | | | 0 | 0.86 | 0.72 |  | 5 | | 0 | 0.63 | 0.83 |  |
| ZcwF07 | 155-175 | 7 | 0 | 0.86 | 0.85 |  | 8 | 0 | 0.84 | 0.85 |  | 8 | | | 0 | 0.79 | 0.86 |  | 6 | | 0 | 0.69 | 0.71 |  |
| ZcwE12* | 189-211 | 10 | 2 | 0.79 | 0.80 |  | 5 | 0 | 0.93 | 0.69 |  | 8 | | | 0 | 0.57 | 0.86 |  | 8 | | 1 | 0.78 | 0.86 |  |
| Mean |  | 7.7 |  | 0.72 | 0.77 |  | 6.7 |  | 0.77 | 0.79 |  | 6.5 | | |  | 0.69 | 0.79 |  | 6.4 | |  | 0.72 | 0.77 |  |

(continue)

| ***Locus*** | **Allele size interval (bp)** | **Argentina** | | | | | | **Uruguay** | | | |  | **Overall** | | |
| --- | --- | --- | --- | --- | --- | --- | --- | --- | --- | --- | --- | --- | --- | --- | --- |
|  |  | **A** | **E** | **Ho** | **He** |  | **A** | **E** | **Ho** | **He** |  | **A** | | **He** |  |
| ZcwB07 | 192-212 | 8 | 0 | 0.94 | 0.81 |  | 8 | 1 | 0.60 | 0.83 |  | 10 | | 0.82 |  |
| Hg8.10 | 200-238 | 13 | 2 | 0.83 | 0.90 |  | 6 | 1 | 0.79 | 0.70 |  | 18 | | 0.88 |  |
| ZcwE04 | 125-141 | 4 | 1 | 0.70 | 0.54 |  | 3 | 1 | 0.33 | 0.54 |  | 8 | | 0.64 |  |
| Pvce* | 128-146 | 6 | 0 | 0.64 | 0.73 |  | 3 | 0 | 0.00 | 0.62 |  | 9 | | 0.77 |  |
| M11a | 173-197 | 8 | 0 | 0.71 | 0.84 |  | 7 | 0 | 0.79 | 0.83 |  | 11 | | 0.82 |  |
| ZcwG04 | 172-212 | 12 | 1 | 1.00 | 0.90 |  | 9 | 0 | 0.71 | 0.86 |  | 17 | | 0.90 |  |
| Pv9 | 193-213 | 6 | 0 | 0.55 | 0.77 |  | 5 | 0 | 0.62 | 0.72 |  | 10 | | 0.83 |  |
| Hg6.3* | 242-258 | 5 | 1 | 0.71 | 0.73 |  | 4 | 0 | 0.50 | 0.54 |  | 8 | | 0.76 |  |
| ZcwF07 | 155-175 | 9 | 0 | 0.80 | 0.83 |  | 5 | 0 | 0.43 | 0.64 |  | 10 | | 0.83 |  |
| ZcwE12* | 189-211 | 8 | 0 | 1.00 | 0.88 |  | 5 | 0 | 0.33 | 0.67 |  | 11 | | 0.87 |  |
| Mean |  | 7.9 |  | 0.79 | 0.79 |  | 5.5 |  | 0.51 | 0.70 |  | 11.2 | | 0.81 |  |

The loci pairs that demonstrated significant LD in each population: Northern Chile: ZcwB07 and ZcwE04, Pvce and M11a, Pvce and Pv9, ZcwE04 and Hg6.3, ZcwG04 and Hg6.3, Pv9 and ZcwF07, ZcwF07 and Hg6.3; Southern Chile: ZcwE04 and Hg8.10, ZcwG04 and Hg8.10, Hg8.10 and Pv9, ZcwF07 and Hg8.10, ZcwE04 and Pvce, Pvce and Hg6.3, Pvce and M11a, Pvce and ZcwF07, ZcwG04 and Pv9; Falkland (Malvinas) Islands: ZcwB07 and Hg8.10, ZcwB07 and ZcwG04, ZcwB07 and M11a, ZcwB07 and ZcwE12, ZcwE04 and Pv9, ZcwE04 and Hg6.3, ZcwE04 and ZcwE12, ZcwG04 and Hg6.3, ZcwG04 and ZcwE12; Argentina: Pvce and ZcwF07, M11a and Hg6.3, M11a and Pv9 and Uruguay: ZcwG04 and Hg8.10, Pvce and M11a, Pvce and Pv9, Pvce and ZcwE12, M11a and Hg6.3, M11a and ZcwE12, ZcwG04 and Pv9, ZcwG04 and Hg6.
