# Supplementary figures and images for: Ancient female philopatry, asymmetric male gene flow, and synchronous population expansion support the influence of climatic oscillations on the evolution of South American sea lion (Otaria flavescens)
Source: PLoS One. 2017 Jun 27;12(6):e0179442. doi: 10.1371/journal.pone.0179442 (PMC5487037; doi:10.1371/journal.pone.0179442)

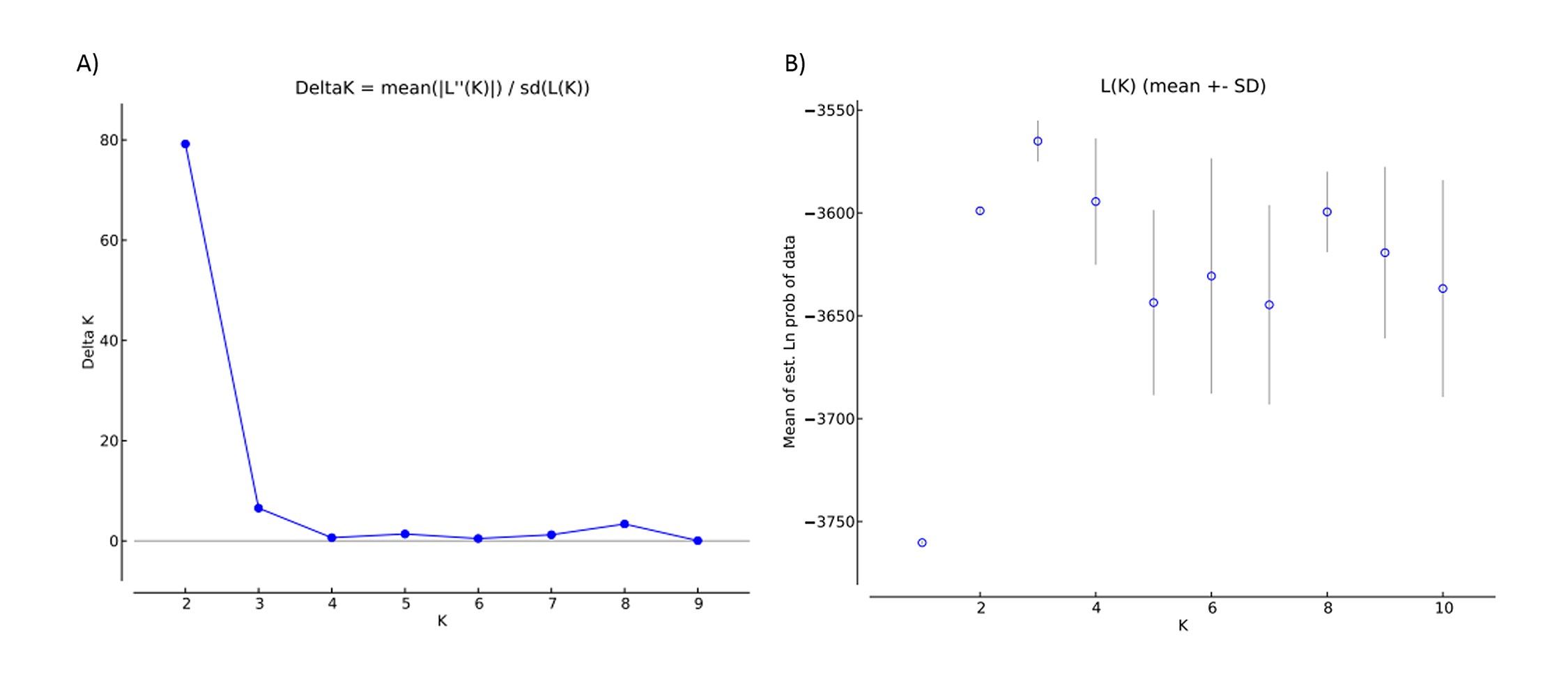

Supplement: S1 Fig — (A) Highest value of (ΔK) = 79.20 on K = 2. (B) Mean of estimated Ln probability of data (± sd) averaging ten runs from K = 1 to K = 10. (TIF) [file pone.0179442.s005.tif]

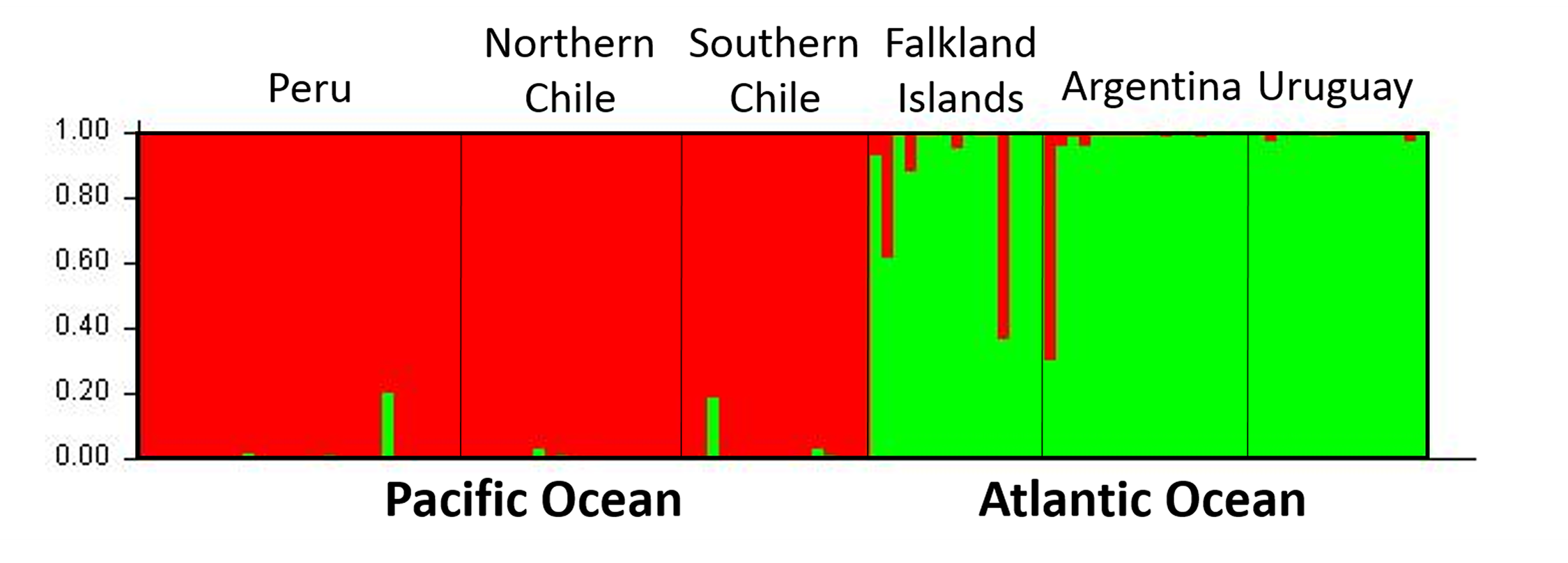

Supplement: S2 Fig — Each bar is one individual and each colour represents the assignment probability of the individual to belong to that genetic cluster. (TIF) [file pone.0179442.s006.tif]

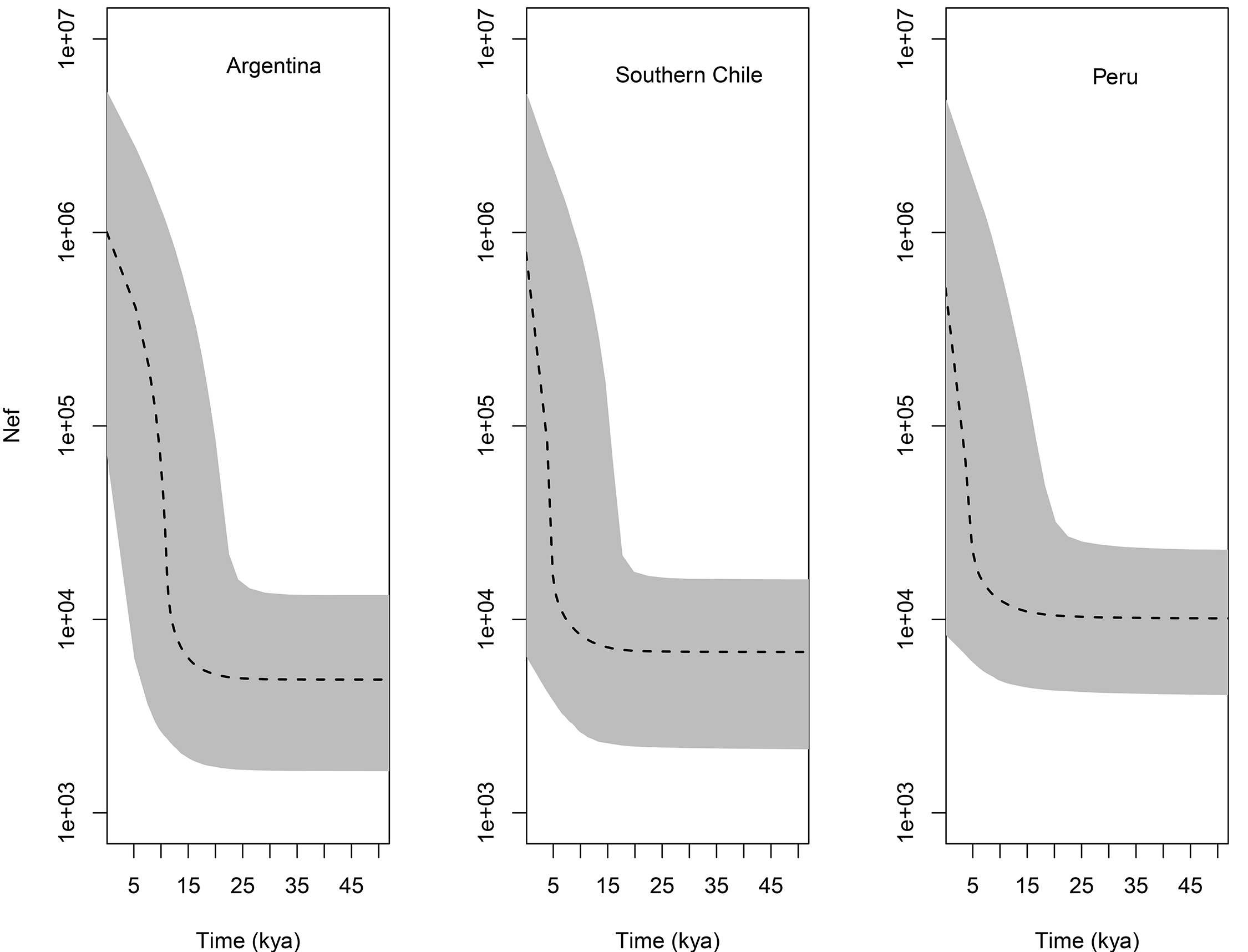

Supplement: S3 Fig — Internal dashed lines are median estimates and thin lines and coloured areas are the 95% Central Posterior Density (CPD) intervals. Nef, effective female population size (log scale), kya, thousands of years ago. (TIF) [file pone.0179442.s007.tif]

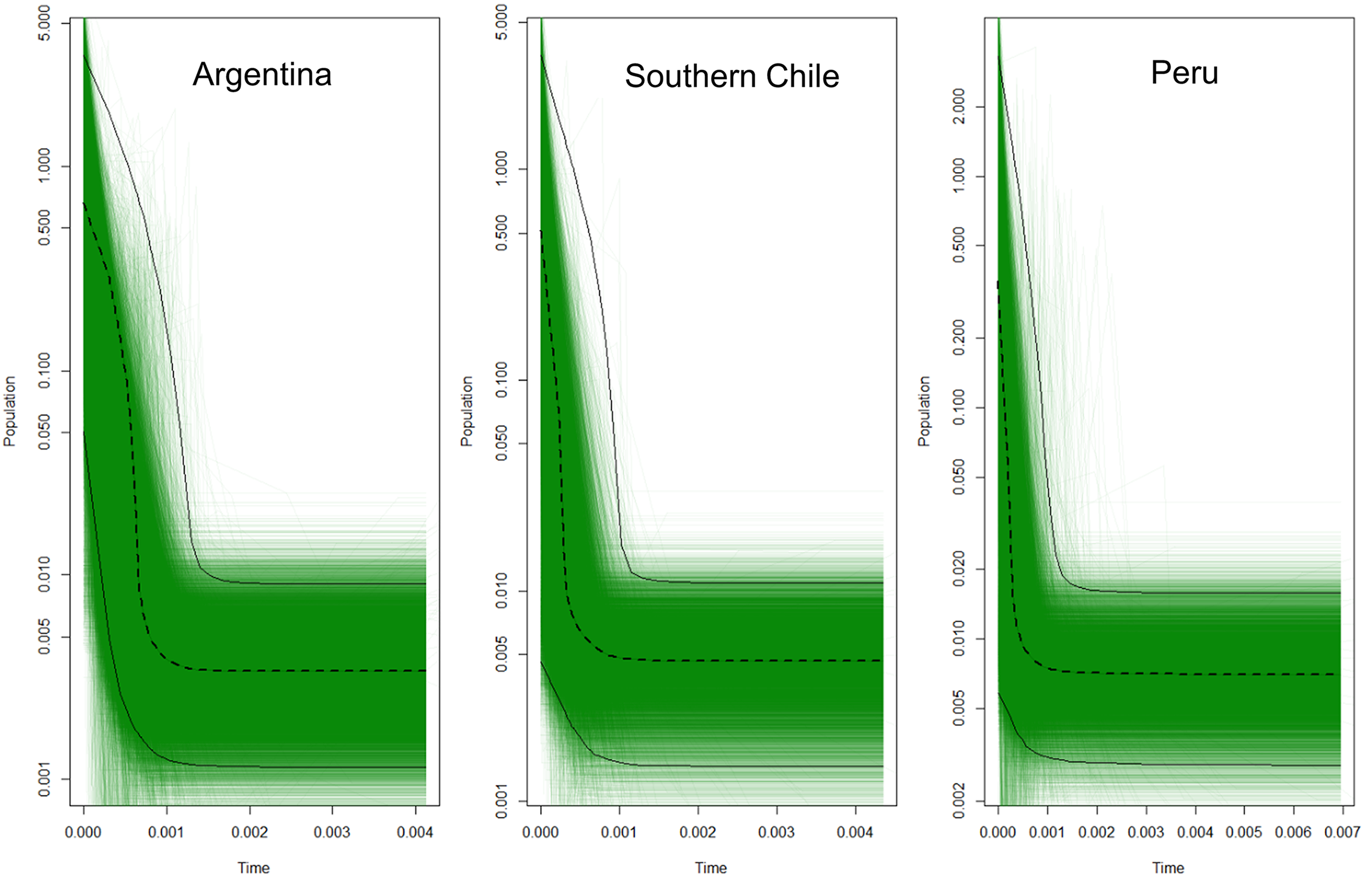

Supplement: S4 Fig — Internal black dashed lines are median estimates and thin lines are the 95% CPD intervals. Thin green lines are the individual population trajectories. Nef, effective female population size (log scale), kya, thousands of years ago. (TIF) [file pone.0179442.s008.tif]
